# Bioinformatics-Facilitated Identification of Novel Bacterial Sulfoglycosidases That Hydrolyze 6-Sulfo-*N*-acetylglucosamine

**DOI:** 10.1021/acsbiomedchemau.4c00088

**Published:** 2024-11-19

**Authors:** Mochen Dong, Zhuoyun Chen, Yuan He, Rémi Zallot, Yi Jin

**Affiliations:** + School of Chemistry, Cardiff University, Cardiff CF10 3AT, United Kingdom; ⊥ Key Laboratory of Synthetic and Natural Functional Molecule, College of Chemistry and Materials Science, Northwest University, Xi’an 710127, P. R. China; ^ Department of Life Sciences, Manchester Metropolitan University, Dalton Building, Chester Street, Manchester M1 5GD, United Kingdom; & Manchester Institute of Biotechnology, University of Manchester, 131 Princess Street, Manchester M1 7DN, United Kingdom; ∇ Department of Chemistry, School of Natural Sciences, Faculty of Science and Engineering, University of Manchester, Oxford Road, Manchester M13 9PL, United Kingdom

**Keywords:** human microbiota, glycan sulfation, glycosyl hydrolase, *N*-acetyl-6-*O*-sulfo-d-glucosamine, genomic enzymology

## Abstract

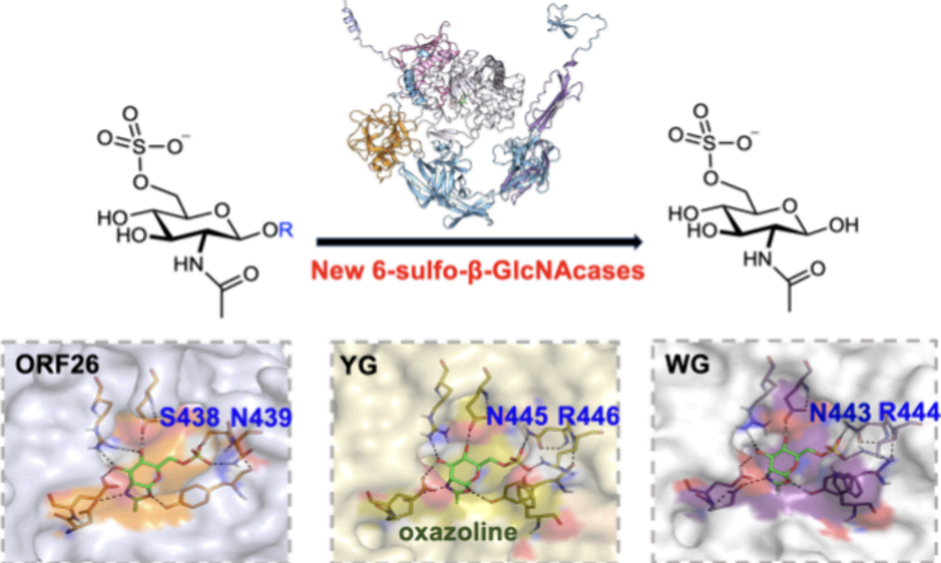

Glycan sulfation is a widespread postglycosylation modification crucial for modulating biological functions including cellular adhesion, signaling, and bacterial colonization. 6-Sulfo-β-GlcNAcases are a class of enzyme that alters sulfation patterns. Such changes in sulfation patterns are linked to diseases such as bowel inflammation, colitis, and cancer. Despite their significance, 6-sulfo-β-GlcNAcases, which cleave β-linked 6-sulfo-*N*-acetylglucosamine (6S-GlcNAc), have been but rarely identified. This scarcity results mainly from the short, diverse, and distinctive sulfate-binding motifs required for recognition of the 6-sulfate group in 6S-GlcNAc in addition to the conserved GH20 family features. In this study, we discovered 6-sulfo-β-GlcNAcases and assigned two novel sulfate-binding motifs by the use of comparative genomics, structural predictions, and activity-based screening. Our findings expand the known microbiota capable of degrading sulfated glycans and add significant enzymes to the tool kit for analysis and synthesis of sulfated oligosaccharides.

Glycan sulfation is a widespread postglycosylation modification that plays an important role in modulating biological function.^1^ For example, the intricate sulfation patterns in extracellular heparan sulfate proteoglycan chains significantly impact cellular adhesion, biological signaling, and dysregulation in these patterns is implicated in various cancers.^2^ Human coronaviruses identify and attach to sulfated *N*-glycans present in the human lung.^3^ Additionally, the negatively charged sulfate group contributes to the colonization of pathogenic and commensal bacteria by mediating bacterial adhesion to mucin *O*-glycans in various sites, including the bronchial airway, lung, and ovarian cyst.^4−6^ The degree of sulfation can modify the physicochemical properties of mucins, which serve as a barrier between human microbiota and epithelium.^7^ Changes in sulfation patterns have been linked to a compromised mucus barrier function, clinically associated with conditions such as inflammatory bowel disease, colitis, Crohn’s disease, carcinoma, and cystic fibrosis.^4,8−10^

Sulfation has been found in glycosaminoglycans (GAGs), decorating *N*-acetylglucosamine (6S-GlcNAc), *N*-acetylgalactosamine (6S-GalNAc and 4S-GalNAc), galactose (3S-Gal, 4S-Gal, and 6S-Gal), and mannose (6S-Man).^3,5,11,12^ In contrast to the extensively identified human and bacterial sugar sulfatases,^13,14^ the availability of glycosyl hydrolases (GHs) capable of directly cleaving sulfated sugars is notably limited.^15^ To date, the exclusive sulfated GlcNAc directly cleavable by GHs is β-linked 6S-GlcNAc, and these GHs are denoted as 6-sulfo-β-GlcNAcases. Three 6-sulfo-β-GlcNAcase belong to the GH20 family, namely BbhII from Gram-positive *Bifidobacterium bifidum* JCM 7004 and JCM 1254,^16^ SGL from Gram-negative *Prevotella* strain RS2,^17^ and Bt4394 from Gram-negative *Bacteroides thetaiotaomicron*(17) were discovered through individual screenings of the respective organisms or their lysate against various substrates, revealing their capability to hydrolyze 6S-GlcNAc in an *exo* fashion. Functional metagenomics was subsequently employed successfully to identify another GH20 *exo*-acting 6-sulfo-β-GlcNAcase, F3-ORF26, from *Phocaeicola dorei*, that selectively cleaves 6S-GlcNAc from screening 24,000 clones.^18^ Recently, the catalytic activity of a novel GH185 family 6-sulfo-β-GlcNAcase (Sp_0475) from *Streptococcus pneumoniae* TIGR4 was identified serendipitously through extensive substrate screening.^19^ This underscores the challenge of identifying novel enzymes with 6-sulfo-β-GlcNAcase activity. Nevertheless, the biocatalysis industry is increasingly seeking to exploit the transglycosylation activity of β-*N*-acetylglucosaminidases for the enzymatic synthesis of well-defined sulfated oligosaccharides and inhibitors, thereby replacing expensive chemical syntheses.^16,20,21^ Successful examples of the use of GH20 variants for the formation of β-thioGlcNAc linkages demonstrate the potential to attach nonhydrolyzable β-thio-6S-GlcNAc to thiosugars and to cysteine residues using 6-sulfo-β-GlcNAcases.^22^*Exo*-acting β-*N*-acetylglucosaminidases that recognize 6S-GlcNAc are also sought for determining precise sulfation sites on glycans during *exo*-glycosidase sequencing.^23,24^

Structural characterization and sequence alignment have revealed that in addition to conserved features shared with GH20 family enzymes, the GH20 6-sulfo-β-GlcNAcases exhibit distinctive patterns around the sulfate-binding motif, which are necessary for recognizing 6-sulfation in 6S-GlcNAc as the substrate. For example, the sulfate recognizing sequence for BbhII follows the pattern of YFP**Q**(X_10_)**W**AC, where Q and W are the residues that identify sulfate. Bt4394 exhibits a pattern of **Q**IPYYI**NR** in which residues Q, N, and R are the residues involved in the sulfate binding. The sulfate binding residues in SGL are identified as C and R in motif YYI**CR**. The structure of human GH20 HexA, reported to possess 6-sulfo-β-GlcNAcase activity, shows that the N and R residues in the YL**NR** motif are involved in 6-sulfate recognition, as for Bt4394. Additionally, a tyrosine residue from a neighboring chain also contributes to sulfate binding following postmaturation (PDB: 2GK1).^25^ The single common feature among these diverse sulfate-binding motifs of the above enzymes, at the sequence level, is the presence of a conserved tyrosine residue (Y underscored) proximate to the sulfate-binding residues. This essential tyrosine donates a hydrogen bond (H-bond) to the *N*-acetyl carbonyl oxygen in 6S-GlcNAc to assist cyclization and subsequently to the ring oxygen of the oxazoline intermediate product. In addition to the varied patterns observed in the sulfate-binding motif, a second challenge for the identification of GH20 β-*N*-acetylglucosaminidases with 6-sulfo-β-GlcNAcase activity is the diversity of sulfate-binding residues, which include not only positively charged amino acids such as arginine but also neutral residues with H-bond donating side chains including glutamine, asparagine, cysteine, tryptophan, and tyrosine as well as waters. These complexities make the prediction of specificity based solely on protein sequence not feasible.

AlphaFold^26^ provides valuable information for relatively accurate overall structural predictions, especially for multidomain proteins that may be hard to crystallize. However, the sulfate-binding motifs in 6-sulfo-β-GlcNAcases are often located within a poorly conserved loop region,^15^ lacking well-defined structural features, and this can reduce the accuracy of prediction. Moreover, employing AlphaFold-predicted structures to identify key sulfate-binding residues in new enzyme families with low sequence and structural similarity poses significant challenges.^17^ Against this discouraging analysis, the integration of bioinformatics approaches^27^ with structural data from traditional structural biology techniques and AI-driven tools such as AlphaFold offers a complementary, efficient strategy for screening metagenomic data for discovery of unknown GH enzymes having 6-sulfo-β-GlcNAcase activity. Our approach has now proved successful in the current study, where we conduct further characterization of the previously discovered 6-sulfo-β-GlcNAcase F3-ORF26, to identify serine as a significant evolutionarily selected residue involved in sulfate binding. Furthermore, by leveraging comparative genomics insights from the Enzyme Function Initiative (EFI) web tools (https://efi.igb.illinois.edu/),^28,29^ and structural data from our prior investigations and AlphaFold predictions, we now identify two additional sulfate-binding sequences capable of recognizing 6-sulfated GlcNAc as a substrate. Collectively, our study reveals that the range of microbiota capable of degrading 6S-GlcNAc from sulfated glycans is broader than had been believed. Moreover, it identifies additional 6-sulfo-β-GlcNAcases that possess valuable potential for analysis and synthesis of sulfated oligosaccharides associated with chronic inflammation and cancer metastasis.^16,30−32^

## Results and Discussion

### Mutagenesis and Kinetic Analysis of F3-ORF26 Reveal Key Sulfate Recognition Residues

**Figure 1:**
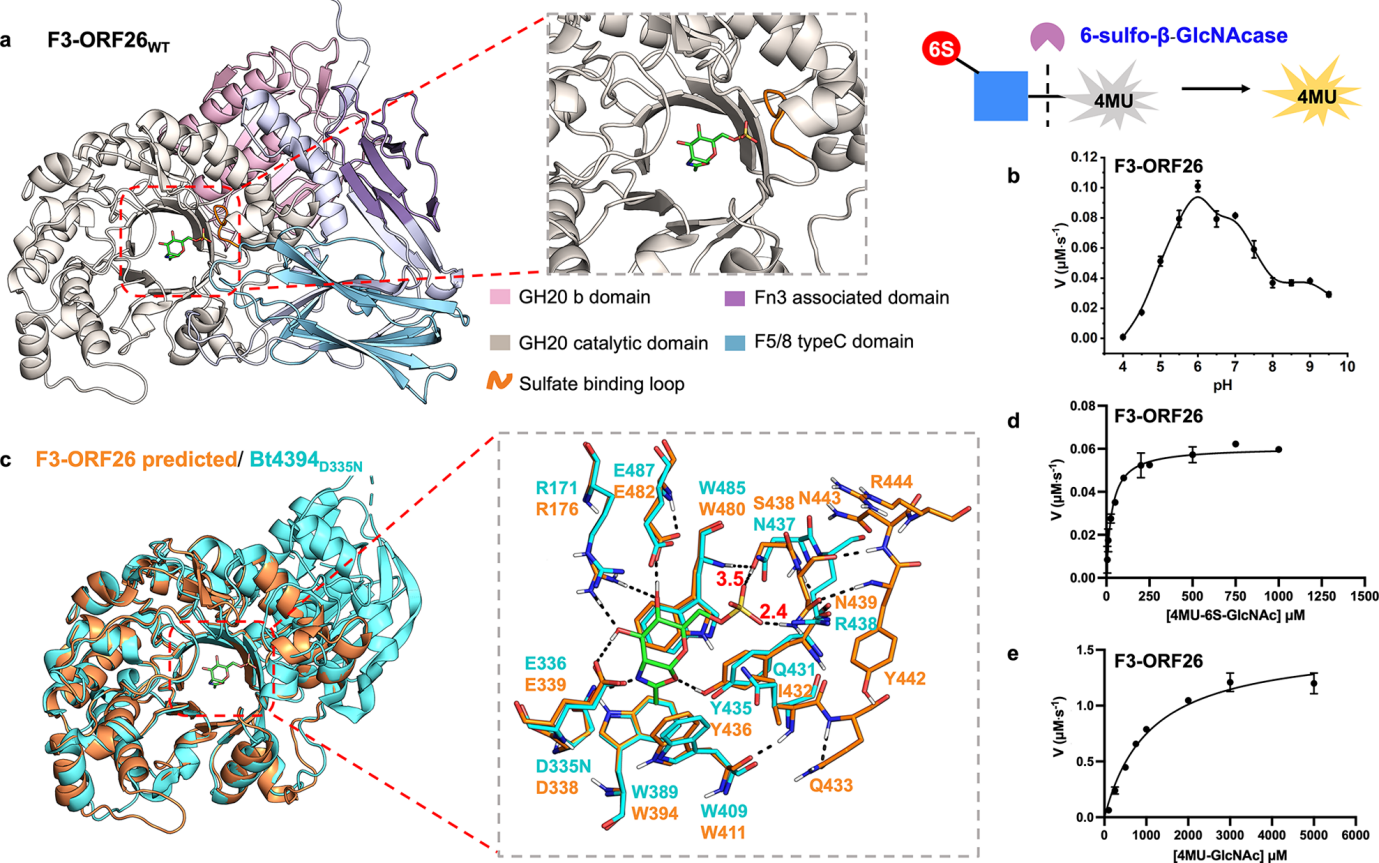
AlphaFold 3-predicted unrelaxed structure of F3-ORF26_WT_ and its biochemical characterization. (a) Unrelaxed apo F3-ORF26 structure predicted by AlphaFold 3 with its multiple domains highlighted in color and with the GH20 catalytic domain presenting a (β/α)_8_ barrel fold aligned to Bt4394_D335N_-6S-NAG-oxazoline structure (PDB: 7DVB, the 6S-NAG-oxazoline is shown as green sticks for clarity; the sulfate-binding loop is highlighted in orange). (b) pH-rate profile for the hydrolysis of 4MU-6S-GlcNAc substrate (500 μM) by F3-ORF26_WT_ (2 nM) across a pH range of 4.0–9.5, with an optimal pH of 6.0. (c) Superposition of the Bt4394_D335N_-6S-NAG-oxazoline structure and that of the GH20 catalytic domain of F3-ORF26. The inset shows residues critical for catalysis and binding of 6S-GlcNAc in the active site. Michaelis–Menten plots for (d) hydrolysis of 4MU-6S-GlcNAc (5 to 1000 μM at pH 6.0) by F3-ORF26_WT_ (1 nM). (e) Hydrolysis of 4MU-GlcNAc (100 to 8000 μM at pH 6.0) by F3-ORF26_WT_ (0.1 μM).

6-Sulfo-β-GlcNAcase F3-ORF26 from *Phocaeicola dorei* (Uniprot ID: A0A4R4I8J5) was predicted to contain multiple domains, including GH20b domain (aa 32–162), GH20 catalytic domain (aa 165–508), Fn3 associated domain (aa 557–613), and F5/8 typeC domain (aa 639–746) (Figure 1a), and has 100-fold higher relative activity toward 4-methylumbelliferyl 6-sulfo-2-acetamido-2-deoxy-β-d-glucopyranoside (4MU-6S-GlcNAc) than 4-methylumbelliferyl 2-acetamido-2-deoxy-β-d-glucopyranoside (4MU-GlcNAc),^18^ but the sulfate-recognizing motif has not been identified. To determine the kinetics, we produced recombinant 6×His-F3-ORF26 from a pJS119 K vector containing the F3-ORF26 gene (residues 22–773) using the NEBExpress *I*^*q*^ strain (NEB).^18^ This recombinant F3-ORF26 protein shows maximum activity at pH 6.0 toward 4MU-6S-GlcNAc in a fluorometric assay (Figure 1b and Figure S1). F3-ORF26 exhibits *k*_cat_ of 61 s^–1^, *K*_M_ of 31 μM, and *k*_cat_/*K*_M_ value of 2.0 × 10^6^ s^–1^ M^–1^ toward 4MU-6S-GlcNAc, which is 286-fold greater than that for 4MU-GlcNAc, further confirming its function as a 6-sulfo-β-GlcNAcase (Figure 1d,e, Figure S2, and Table 1).

**Table 1:** Kinetic Parameters for the Hydrolysis of 4MU-6S-GlcNAc and 4MU-GlcNAc by F3-ORF26, WG Enzyme, YG Enzyme, and Their Variants^a^

protein	substrate/variants	*K*_**M**_**(μM)**	*k*_**cat**_**(s**^**–1**^**)**	*k*_**cat**_**/***K*_**M**_(s^–1^ M^–1^)	**relative activity**	**pH**
Bt4394	wild-type (4MU-6S-GlcNAc)	39 ± 4	25.8 ± 0.7	(7 ± 2) × 10^5^	100%	5.5
	wild-type (4MU-GlcNAc)	2183 ± 189	2.9 ± 0.1	(1.3 ± 0.5) × 10^3^	0.19%	
Bt4394_Q431W,I432G_	4MU-6S-GlcNAc	194 ± 16	12.0 ± 0.3	(6 ± 2) × 10^4^	8.6%	
	4MU-GlcNAc	39820 ± 3738	3.1 ± 0.2	77 ± 62	0.01%	
Bt4394_Q431Y,I432G_	4MU-6S-GlcNAc	546 ± 37	31.6 ± 0.7	(6 ± 2) × 10^4^	8.6%	
	4MU-GlcNAc	15735 ± 2534	3.7 ± 0.4	(2 ± 1) × 10^2^	0.03%	
WG	wild-type (4MU-6S-GlcNAc)	5.1 ± 0.3	39.9 ± 0.6	(8 ± 2) × 10^6^	100%	6.0
	wild-type (4MU-GlcNAc)	2706 ± 344	11.6 ± 0.5	(4 ± 2) × 10^3^	0.05%	
	W437F	8.0 ± 0.8	63 ± 2	(8 ± 3) × 10^6^	100%	
	W437A	17 ± 2	45 ± 1	(2.6 ± 0.3) × 10^6^	33%	
	W437Q	20 ± 3	48 ± 2	(2.4 ± 0.7) × 10^6^	30%	
	G438I	32 ± 3	39 ± 1	(1.2 ± 0.3) × 10^6^	15%	
	N443D	56 ± 6	69 ± 4	(1.2 ± 0.7) × 10^6^	15%	
	R444A	47 ± 6	62 ± 2	(1.3 ± 0.3) × 10^6^	16%	
YG	wild-type (4MU-6S-GlcNAc)	12 ± 1	76 ± 2	(6 ± 2) × 10^6^	100%	6.0
	wild-type (4MU-GlcNAc)	2209 ± 355	11.7 ± 0.7	(5 ± 2) × 10^3^	0.08%	
	Y439F	9.6 ± 0.7	69 ± 1	(7 ± 1) × 10^6^	117%	
	Y439A	10.7 ± 0.8	30.2 ± 0.5	(2.8 ± 0.6) × 10^6^	47%	
	Y439Q	24 ± 2	44 ± 1	(1.8 ± 0.5) × 10^6^	30%	
	G440I	22 ± 1	42.8 ± 0.7	(1.9 ± 0.7) × 10^6^	32%	
	N445D	130 ± 14	98 ± 3	(8 ± 2) × 10^5^	13%	
	R446A	90 ± 9	97 ± 3	(1.1 ± 0.3) × 10^6^	18%	
F3-ORF26	wild-type (4MU-6S-GlcNAc)	31 ± 3	61 ± 2	(2.0 ± 0.5) × 10^6^	100%	6.0
	wild-type (4MU-GlcNAc)	985 ± 247	6.9 ± 0.7	(7 ± 3) × 10^3^	0.35%	
	Q443E	33 ± 2	64.1 ± 0.8	(2.0 ± 0.3) × 10^6^	100%	
	S438A	944 ± 94	98 ± 4	(1.0 ± 0.4) × 10^5^	5%	
	N439D	1987 ± 195	40 ± 2	(2 ± 1) × 10^4^	1%	
	Y442F	33 ± 2	69 ± 1	(2.1 ± 0.6) × 10^6^	105%	
	N443D	139 ± 16	64 ± 2	(5 ± 1) × 10^5^	23%	
	R444A	50 ± 6	82 ± 3	(1.6 ± 0.4) × 10^6^	80%	

a Activities of all variants were measured using 4MU-6S-GlcNAc as substrate.

When superimposed with the 6S-NAG-oxazoline-bound intermediate structure of Bt4394 (PDB: 7DVB), the AlphaFold 3-predicted GH20 catalytic domain of F3-ORF26 aligns very well with that of Bt4394, with an overall RMSD of 0.859. In this predicted structure, catalytic diad D338-E339 closely resembles the catalytic residue pair D335-E336 in Bt4394, adopting a catalytically competent conformation for the substrate-assisted mechanism. Additionally, other conserved residues around the active site in the GH20 family align well with those in the Bt4394-oxazoline intermediate complex (Figure 1c). Although sequence alignment for F3-ORF26 with other 6-sulfo-β-GlcNAcases suggested that either Q433, S438, or N439 in the **Q**_433_FLYF**S**_438_**N**_439_ or N443 and R444 in Y_442_**N**_**443**_**R**_**444**_ could potentially form the sulfate binding loop,^15^ structure alignment indicated that S438 and N439 in F3-ORF26 are more likely to be involved in sulfate recognition. These residues could donate two potential H-bonds (3.5 and 2.4 Å, respectively) to the sulfate oxygens, given that the predicted apo F3-ORF26 structure is unrelaxed with residue-specific confidence metrics, the predicted value of the local distance difference test (pLDDT) above 90 (Figure 1c).^34^ To resolve the ambiguity surrounding the sulfate-binding motif in both sequence-based and structure-prediction-based approaches, we targeted the crystal structure of F3-ORF26. Initial crystallization screening was performed using a commercial Crystal HT Screen, SaltRx HT Screen, and PEG/Ion HT Screen (Hampton Research). Although several hit conditions were obtained after one month, extensive optimization efforts over several additional months did not improve the resolution beyond 6 Å.

To further validate the contribution of key residues within the sulfate recognition site (QFLYF**S**_438_**N**_439_PTYN_443_R_444_) and verify the AlphaFold prediction, we individually mutated all potential sulfate binding residues identified by multiple sequence alignments (MSA) in F3-ORF26 by site-directed mutagenesis and subsequently performed kinetics measurements. The variants S438A and N439D exhibited diminished affinity (*K*_M_) by 30- and 66-fold, respectively, with corresponding reductions in *k*_cat_/*K*_M_ of 20- and 100-fold, whereas the *K*_M_ values for N443D and R444A were reduced only by 4.6- and 1.6-fold (Figure S2, Table 1). This highlights S438 and N439 as key residues in sulfate recognition, while N443 and R444 are second-shell residues, as predicted. The relative activity for Q433E is 100%, indicating that Q433, previously thought to be involved in sulfate recognition,^15^ is not essential for this function. These results strongly support the viability of the AlphaFold three-predicted structure of F3-ORF26 (Figure 1c).

**Figure 2:**
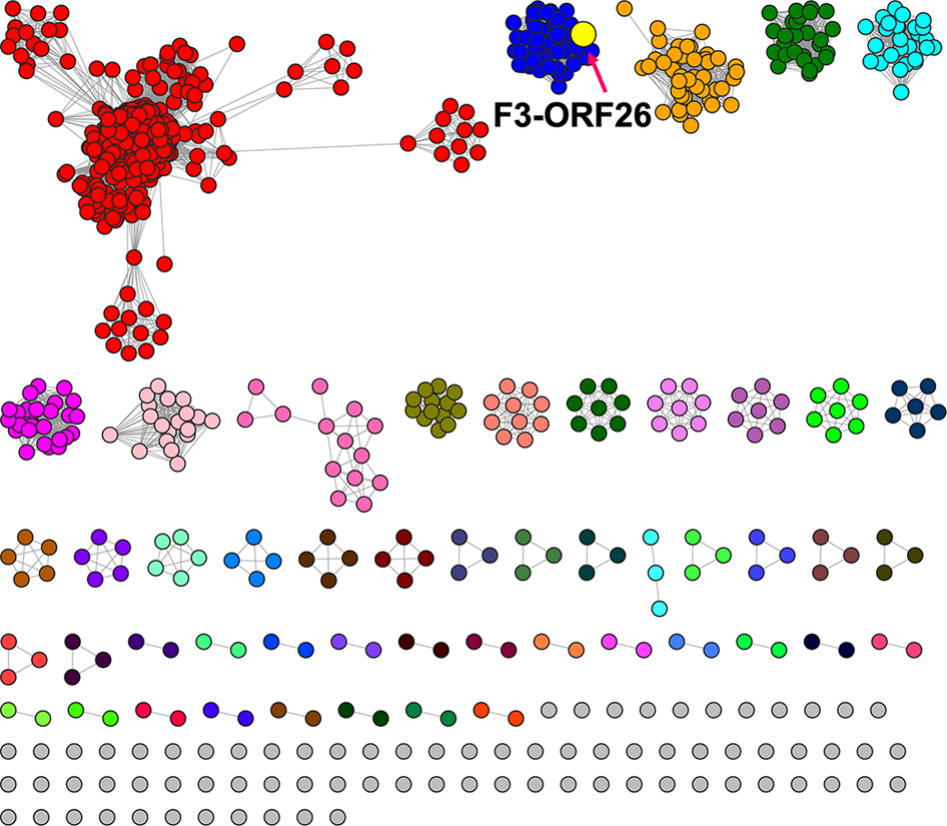
F3-ORF26 6-sulfo-β-GlcNAcases (highlighted as a yellow dot) mapped on the Uniport SSN created for the GH20 domain. The AST was increased to 250 to finally separate the subclusters that correspond to differences in the sequence similarity.

To facilitate the discovery of more potential 6-sulfo-β-GlcNAcases with the same sulfate recognition pattern as in F3-ORF26, we generated Sequence Similarity Networks (SSNs) using the EFI web tools.^28,29^ Previously, we observed that F3-ORF26 appears in the largest (also the first) cluster of the full-resolution SSN network for all GH20 domains,^15^ indicating that the GH20 domain of F3-ORF26 is more similar in sequence to other GH20 family enzymes than to Bt4394, BbhII, and SGL. Thus, this time, we created a “focused” SSN by submitting the GH20 domain sequence of F3-ORF26 for BLAST against the latest Uniprot database for the Pfam-defined protein family PF00728, which is for the GH20 domain. A higher alignment score threshold (AST) was required to isolate the cluster of F3-ORF26 from other GH20 domain sequences. A stepwise increase in the AST value from 178 to 202 (Figure S3a,b) and finally to 250 (Figure 2, Figure S3c) was used. It enables the separation of F3-ORF26 and closely related sequences from those that do not possess the identified sulfate recognition pattern. All sequences in the F3-ORF26 protein cluster shared at least 55% of their identity. Subsequently, an MSA was generated for sequences from the F3-ORF26 cluster, identifying the absolute conservation of residues for catalysis (Figure S4). This cluster included 59 sequences, all exhibiting the ‘SN’ pattern as the sulfate recognition residues, suggesting that they all are 6-sulfo-β-GlcNAcases.

### Identifying New Sulfate-binding Motifs

**Figure 3:**
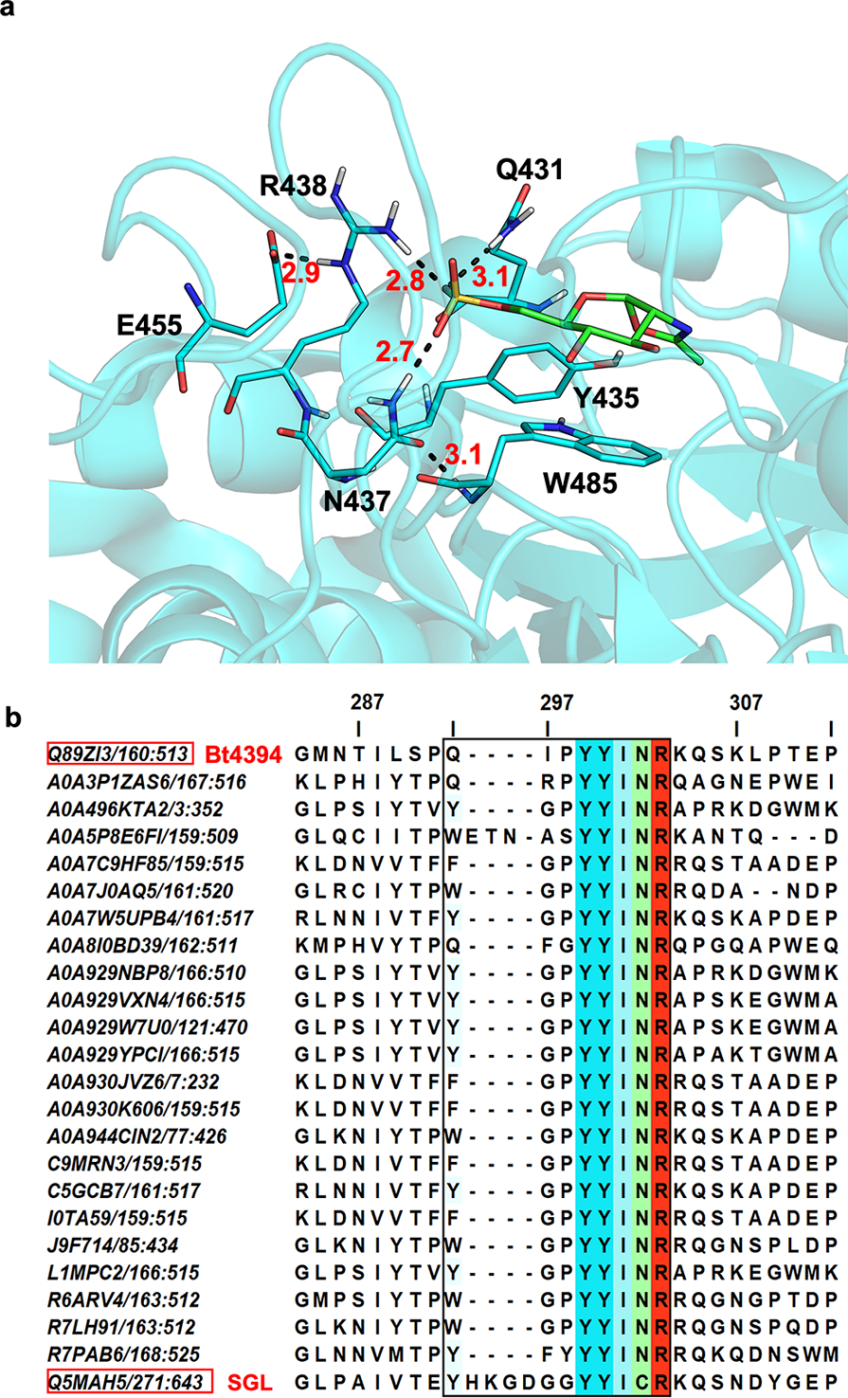
(a) Structure of Bt4394_D335N_-6S-NAG-oxazoline intermediate complex (PDB: 7DVB, cyan), showing that the side chain of Q431 appears more exposed to the solvent and coordinated with one sulfate oxygen. H-bond distances are for donor-to-acceptor in Å. (b) The multiple sequence alignment (MSA) generated by Clustal Omega shows the portion of sequences around the sulfate-binding motif (boxed) from the SSN cluster containing SGL. The alignment indicates that NR residues are more conserved. Each sequence is identified by its UniProt ID from the GH20 domain (PF00728). The color scheme is based on JalView Clustal coloring, with a default conservation setting of 30. The top sequence, Q89ZI3, corresponds to Bt4394 and is used as a reference, while the bottom sequence, Q5MAH5, corresponds to SGL.

After careful inspection of the sulfate binding environment in Bt4394 in the structure of Bt4394_D335N_-6S-NAG-oxazoline complex (PDB: 7DVB), we noticed that, unlike N437 and R438 in the sequence **Q**_**431**_IPYYI**N**_**437**_**R**_**438**_, which are well coordinated by second-shell residues W485 and E455 (Figure 3a), the side chain of Q431 appears more exposed to the solvent and interacts only with one sulfate oxygen, thus offering greater flexibility. Intrigued by this observation, we questioned whether this glutamine could be replaced by other H-bonding amino acids while retaining 6-sulfo-β-GlcNAcase activity.

To evaluate the prevalence of sequences that have the identified binding signature, we created SSNs of GH20 family proteins (PF00728 for the GH20 domain only) using the Pfam Protein family database, searching for any moiety with YYI**NR**. In contrast to the UniRef90 SSN previously used, a full-resolution SSN was generated and updated with the most recent available information for exploration and analysis of the residues from the catalytic domain. When the AST is 130, corresponding to ±60% sequence identity, we found two clusters containing SGL and Bt4394 (Figure S5) with sequences corresponding to YYI**NR** that aligned with the respective region in the Bt4394 sequence (Figure 3b). The MSA alignment showed that the most common amino acids preceding YYI**NR** are tryptophan W and tyrosine Y, which have H-bond donating ability.

**Figure 4:**
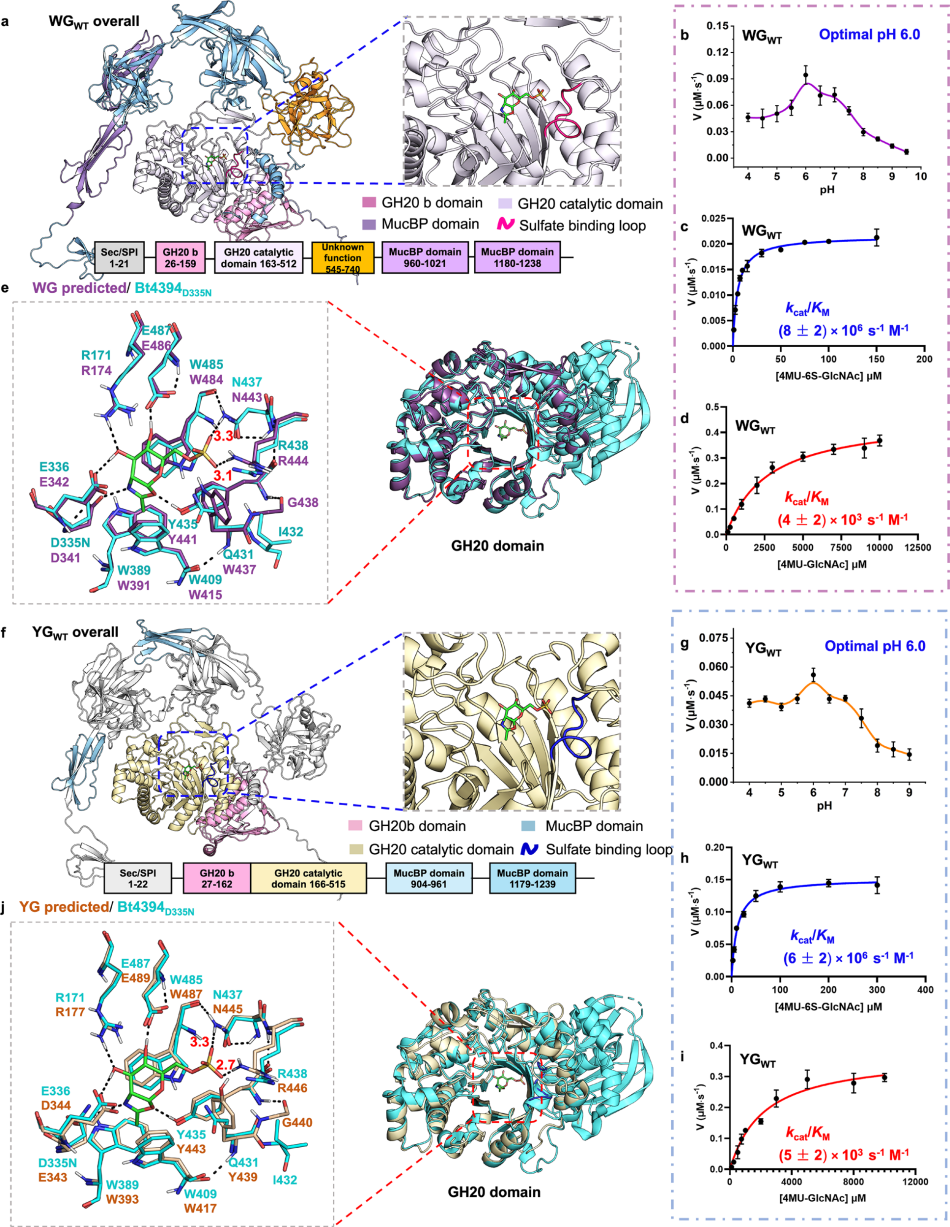
AlphaFold 3-predicted unrelaxed structure of WG and YG enzymes and their biochemical characterization. (a) Predicted unrelaxed structure of full-length WG_WT,_ with its multiple domains highlighted in color and with the GH20 catalytic domain aligned to the Bt4394_D335N_-6S-NAG-oxazoline structure (the sulfate-binding loop highlighted in magenta). (b) pH-rate profile for the hydrolysis of 4MU-6S-GlcNAc (50 μM) substrate by WG_WT_ (2 nM) over a pH range of 4.0–9.5, with an optimal pH of 6.0. Michaelis–Menten plots for (c) the hydrolysis of 4MU-6S-GlcNAc (1–150 μM) by WG_WT_ (0.5 nM) and (d) the hydrolysis of 4MU-GlcNAc (100–10000 μM) by WG_WT_ (40 nM) at pH 6.0. H-bond distances are for donor-to-acceptor in Å. (e) Superposition of the predicted GH20 domain structure of WG_WT_ (purple) and the structure of the Bt4394_D335N_-6S-NAG-oxazoline intermediate (cyan). (f) Predicted unrelaxed structure of full-length YG_WT_, with the GH20 catalytic domains aligned with the Bt4394_D335N_-6S-NAG-oxazoline intermediate structure (PDB: 7DVB, only the 6S-NAG-oxazoline is drawn as green sticks for clarity). The sulfate-binding loop is highlighted in dark blue. (g) pH-rate profile for the hydrolysis of 4MU-6S-GlcNAc (100 μM) substrate by YG_WT_ (1.1 nM) over pH range of 4.0–9.0 with an optimal pH of 6.0. Michaelis–Menten plots for (h) the hydrolysis of 4MU-6S-GlcNAc (2.5 μM to 300 μM) by YG_WT_ (2 nM) and (i) the hydrolysis of 4MU-GlcNAc (100 μM to 10000 μM) by YG_WT_ (31.8 nM) at pH 6.0. (j) Superposition of the predicted unrelaxed GH20 domain structures of apoYG_WT_ (salmon) and the Bt4394_D335N_-6S-NAG-oxazoline intermediate complex (cyan). The inset emphasizes essential residues critical to the catalysis and binding of 6S-GlcNAc in the active site.

In order to explore whether these enzymes can still be efficient 6-sulfo-β-GlcNAcases, we identified a full-length gene containing ‘**WG**PYYI**NR**’ (Uniprot ID R6ARV4, “WG enzyme” hereafter) from *Prevotella* sp. CAG:5226 strain and another containing ‘**YG**PYYI**NR**’ residues (Uniprot ID: L1MPC2, “YG enzyme” hereafter) from *Alloprevotella sp*, both of which are only noted as β-*N*-acetylhexosaminidase without further characterization. These two sequences were chosen because they exhibit a lower sequence similarity for the GH20 domain when aligned with the wild-type Bt4394. Both full-length WG and YG proteins contain a signal peptide, indicating that they function as secreted proteins. They both have multiple domains, including the beta-hexosaminidase bacterial type N-terminal domain, the GH20 catalytic domain, and accessory domains such as mucin binding protein domains (MucBP). However, we did not identify any 6S-GlcNAc-specific carbohydrate-binding modules (CBMs) similar to the CBM32 in BbhII through sequence or structure alignment (Figure 4a,f).^34^

We sought to characterize kinetically whether WG and YG enzymes are 6-sulfo-β-GlcNAcases, the genes of the 6×His-tagged WG and YG in the pET23 vector expressed in a BL21(DE3) Star *E. coli* strain. Both recombinant WG and YG proteins show maximum activity at pH 6.0 toward 4MU-6S-GlcNAc in a fluorometric assay (Figure 4b,g). The *k*_cat_/*K*_M_ values are (8 ± 2) × 10^6^ and (6 ± 2) × 10^6^ s^–1^ M^–1^, respectively, 1 order of magnitude higher than that of Bt4394_WT_ (Table 1). Their specificity toward the sulfated substrate is ∼2000-fold greater than the nonsulfated substrate, confirming they function as 6-sulfo-β-GlcNAcases (Figure 4c,d,h,i, Table 1).

### Q Is Not as Conserved as NR Residues in the Sulfate-Binding Motifs

We initially used AlphaFold 2 for structure prediction for the WG and YG enzymes (Figure S6). Overall, the N-terminal GH20b domain and GH20 catalytic domain of the predicted structures of WG and YG enzymes align well with their counterparts (residues 163–512) from Bt4394. In the active site, key catalytic residues, including the polarizing residue D341 and the general acid/base residue E342 in WG enzyme (D343 and E344 in YG enzyme), aligned well with D335N and E336 in Bt4394. Additionally, R174 and E486 in WG (R177 and E489 in YG), which coordinate the 3′,4′-OH groups, overlaid well with R171 and E487 in Bt4394 (Figure 4e,j). Importantly, AlphaFold 2 predicted that the highly conserved sulfate-binding residues N and R in WG and YG enzymes would function similarly to N437 and R438 in Bt4394, recognizing the sulfate.

To test this, we generated variants of both WG and YG enzymes, replacing N443/445 with D or R444/446 with A. These two variants exhibited diminished affinity, with approximately 10-fold and 9-fold increases in *K*_M_, respectively, leading to reductions in *k*_cat_/*K*_M_ and retaining only 13% to 18% of the corresponding wild-type enzyme activities (Table 1, Figures S7e,f and S8e,f). This demonstrates the significant role of these residues in sulfate recognition through both H-bonding and electrostatic interactions.

### The Conformations of the Sulfate-Binding Motif Predicted by AlphaFold 2 and 3 Are Different

**Figure 5:**
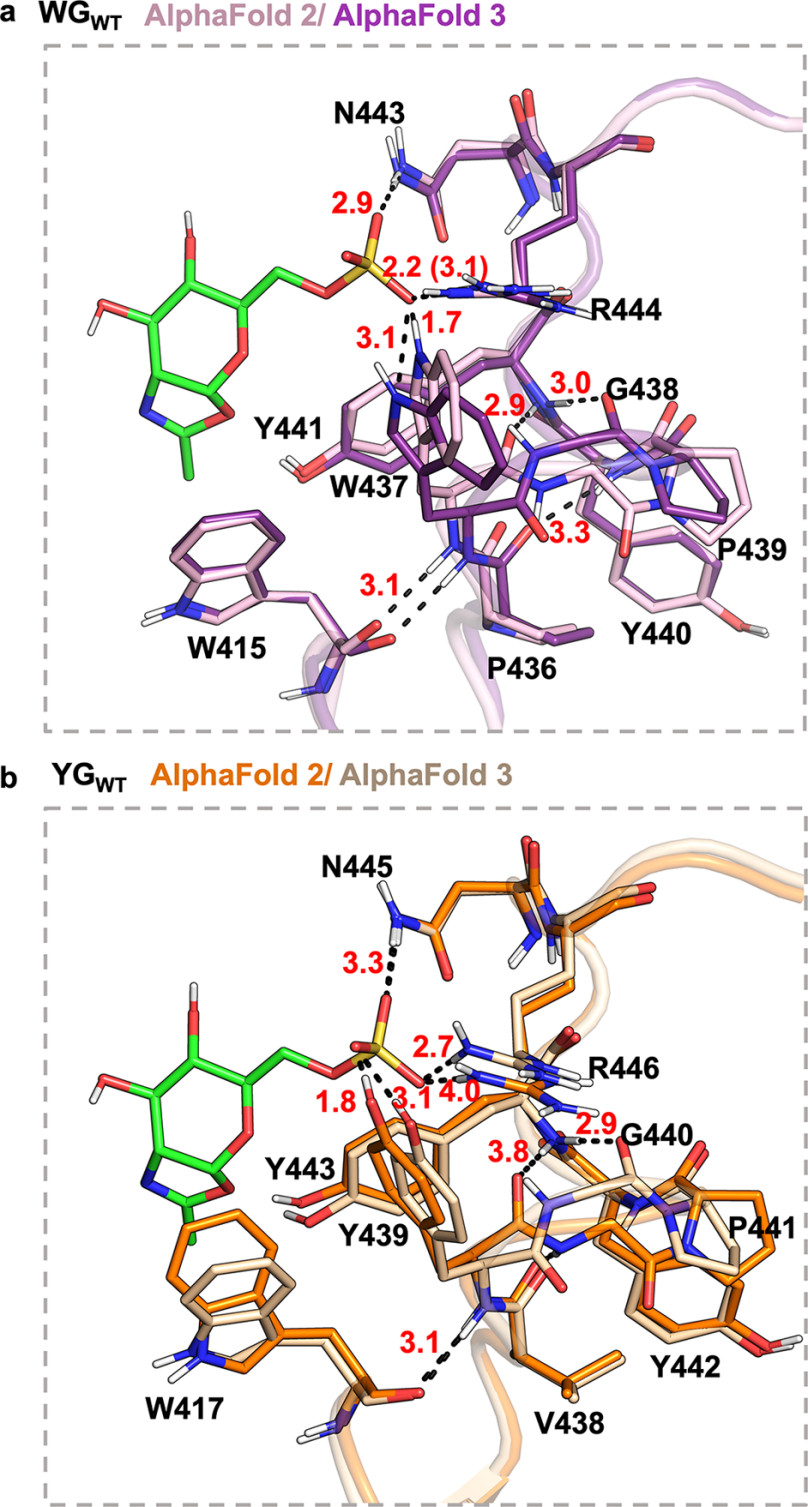
Comparison of the sulfate-binding motif in WG_WT_ and YG_WT_ structures predicted by two versions of AlphaFold, with their active sites aligned to the Bt4394_D335N_-6S-NAG-oxazoline intermediate structure (PDB: 7DVB, only the 6S-NAG-oxazoline ligand in green is shown for clarity). The residues around the sulfate binding motif of unrelaxed WG_WT_ structures predicted by (a) AlphaFold 2 and AlphaFold 3 are shown to highlight differences in conformations for the W_437_-G_438_-P_439_ region. Similarly, the residues around the sulfate binding motif of unrelaxed YG_WT_ structures predicted by (b) AlphaFold 2 and AlphaFold 3 show differences in the conformations for the Y_439_-G_440_-P_441_ region. H-bond distances are for donor-to-acceptor in Å.

While we were carrying out this project, AlphaFold 3 was launched in May 2024 as an upgrade to AlphaFold 2. Given the significant advancements of AlphaFold 3 in predicting both side chain and backbone conformations,^33,35^ we took the opportunity to compare the predicted apoenzyme structures generated by both versions of AlphaFold. We found that the backbone conformations in the sulfate-binding loop of WGP and YGP residues differ between the AlphaFold 2 and AlphaFold 3-predicted models for both WG and YG enzymes (Figure 5). This observation suggests potential flexibility and conformational uncertainty in this region. For the WG enzyme, in the AlphaFold 2-predicted structure, the backbone carbonyl oxygen and amide NH of W437 form a 2.9 Å H-bond with the backbone NH of Y441 and a 3.1 Å H-bond with the backbone carbonyl oxygen of W415, similar to the H-bonding pattern in Bt4394. However, in the AlphaFold 3 structure, while the 3.1 Å H-bond between amide NH of W437 and the backbone carbonyl of W415 remains, the peptide bond between W437 and G438 flips nearly 180°, so the G438 backbone carbonyl accepts a 3.0 Å H-bond from the backbone NH of Y441, and one extra 3.3 Å H-bond is formed between Y440 backbone NH and the carbonyl of P436. This extra H-bond may potentially lower the energy of the sulfate-binding loop and increase its stability. Such a rearrangement of the W_437_-G_438_-P_439_ region also changes the Ramachandran angles of W437. When the Bt4394_D335N_-6S-NAG-oxazoline intermediate structure was aligned with the AlphaFold 2 structure, the separation of the W437 ring-nitrogen and the sulfate oxygen is only 1.7 Å, too short for a H-bond, thus indicating a van der Waals steric clash (Figure 5a). This distance in the AlphaFold 3 structure is more acceptable, where the separation is 3.1 Å, although the O···H–N angle between the indole NH group of W437 and the sulfate oxygen is only 113°, not a suitable conformation for effective H-bonding (Figure 5b). Although these distances and angles could change by rotating the W437 side chain, both cases suggest that W437 may not participate in 6-sulfate binding. Similar to the WG enzyme, the AlphaFold 3-predicted structure of the YG enzyme shows a peptide bond flip between Y439 and G440 in the Y_439_-G_440_-P_441_ region (Figure 5a,b). Additionally, an extra 2.9 Å H-bond is also formed between the NH of Y443 and the carbonyl oxygen of G440. However, different from the WG enzyme, the Y439 side chain–OH could potentially donate a 3.1 Å H-bond to the sulfate oxygen without much clashing (Figure 5b). Given that the predictions by AlphaFolds 2 and 3 did not consider the presence of 6-GlcNAc or 6S-NAG-oxazoline, the reduced van der Waals steric clashes in the AlphaFold 3 prediction represent a remarkable improvement over AlphaFold 2.

### Solvent-Exposed Residues May Not Be Always Involved in Sulfate Binding

Our attempts to crystallize full-length multidomain WG and YG enzymes were unsuccessful. We therefore tried to generate truncated enzymes containing only the GH20 catalytic domain, aiming to cocrystallize them with 6S-GlcNAc better to define the sulfate-binding motif. However, despite several rounds of expression optimization (Supporting Information), we could not obtain soluble protein sufficient for crystallization as most of the truncated protein formed inclusion bodies when expressed in *E. coli* (Supporting Information). Thus, to validate whether these solvent-exposed residues, such as W437 in the WG enzyme and Y439 in the YG enzyme, participate in sulfate recognition and examine the correctness of the predicted structures, we generated a series of variants. For the WG enzyme, WG_W437F_ and YG_Y439F_ show similar *K*_M_ and activity to their corresponding wild-type enzymes (Table 1, Figures S7a and S8a), suggesting that W437 and Y439 do not clash with the sulfate or participate in binding by donating the H-bond to the sulfate directly. In this regard, neither the structure predicted by AlphaFold 2 nor the one predicted by AlphaFold 3 exhibits the correct side chain conformation for the solvent-exposed residues involved in sulfate binding. WG_W437A_ and YG_Y439A_ variants show a 2 to 3-fold decrease in activity suggesting a more steric and rigid amino acid flanking the sulfate group may provide some advantage to the substrate binding (Figures S7b and S8b). We also generated WG_W437Q_ and YG_Y439Q_ variants, where tryptophan and glutamine are substituted by glutamine, seen as a key H-bond donor for sulfate-coordinating Q431 in Bt4394, with the hope of seeing the same or improved binding. However, both show a 2 to 4-fold increase in *K*_M_ (Table 1, Figures S7c and S8c). Combined with the double conformations of the WGP and YGP_loop_ with the change of Ramachandran angles of W437 in the two predicted structures by AlphaFold 2 and 3, the role of Q431 as a H-bond donor and a recognition residue for the sulfate in Bt4394 may be facilitated by the bulky I432 side chain, providing a less flexible part of the sulfate-binding motif (Figure 4e,j). The change of Ramachandran angles of W437 between AlphaFold 2 and 3 predicted structures could be attributed to the flexibility introduced by G438.^35^

To test this, we mutated G438 to isoleucine in both WG and YG enzymes and found that both exhibited only 15% and 32% of wild-type activity: *K*_M_ increases of 6 and 2-fold, respectively, indicating diminished binding. In contrast, the more rigid and bulkier I432 in Bt4394 may stabilize neighboring residues in the loop and allow Q431 to be appropriately oriented and coordinated with the sulfate oxygen (Table 1, Figure 4e,j, Figures S7d and S8d). Testing this hypothesis further, we generated double-mutation variants Bt4394_Q431W, I432G_ and Bt4394_Q431Y, I432G_ for which AlphaFold 2 and 3 again provided different predictions (Figure S9). Each of these two variants has an increased *K*_M_ value, 194 ± 16 and 546 ± 37 μM, respectively, compared to the Bt4394_WT_*K*_M_ (39 ± 4 μM), and both have *k*_cat_/*K*_M_ of (6 ± 2) × 10^4^ s^–1^ M^–1^, retaining only 9% of the activity of Bt4394_WT_ (Figure S10, Table 1). This illustrates that the sulfate-binding sequence **Q**_**431**_**I**PYYI**N**_**437**_**R**_**438**_ in Bt4394 is better-tuned for sulfate binding than **W/Y**_**431**_**G**PYYI**N**_**437**_**R**_**438**_: the extra H-bond between Q431 and the sulfate, assisted by *I*432, contributes an additional −6.1 kJ mol^–1^ of binding energy for catalysis. However, it is interesting to observe that WG and YG wild-type enzymes with the sequence **W/Y**GPYYI**NR** exhibit a 10-fold higher *k*_cat_/*K*_M_ (8 × 10^6^ and 6 × 10^6^ s^–1^ M^–1^, respectively) compared to wild-type Bt4394 (7 × 10^5^ s^–1^ M^–1^). This suggests that other factors may contribute to this rate difference, such as the presence of accessory domains in WG and YG, differences in protein dynamics, and the second-shell coordination to the sulfate-binding residues.

## Conclusions

The modification of sulfoglycans occurs in various mammalian systems. 6-Sulfo-β-GlcNAcases enable microbes to access sulfated glycans by selectively releasing 6S-GlcNAc from host oligosaccharides, offering an alternative strategy to sulfatases. The identification of these 6-sulfo-β-GlcNAcases indicates the location and environment of the bacteria that produce them. These enzymes also hold great potential for preparing *O*-linked and *S*-linked oligosaccharides containing 6S-GlcNAc as well as glycopeptides and proteins decorated with 6S-GlcNAc.

However, there are two challenges. The easier challenge is assigning key but short and less conserved sulfate-binding motifs in 6-sulfo-β-GlcNAcases discovered through mass screening methods, such as functional metagenomics or laborious substrate screening. Structure prediction tools such as AlphaFold can be instrumental in this process. We successfully assigned sulfate-recognizing motifs for the novel 6S-GlcNAcase F3-ORF26 from *Phocaeicola dorei* using this approach. However, given the current prediction accuracy, especially for proteins from new families,^19^ the AI-predicted binding sites must be verified through mutagenesis studies.

The more difficult challenge is the efficient discovery of more of these enzymes and correctly assigning their short sulfate binding motif, given the difficulties in obtaining high-quality protein crystals for many of these long multidomain proteins via traditional X-ray crystallography. By using existing GH20 6-sulfo-β-GlcNAcase structures as a starting point and diversifying the sequence search for other possibilities around the sulfate binding motif, we combined bioinformatics data from the Enzyme Function Initiative (EFI) web tools with AI structure predictions from AlphaFold models. Through this systematic approach, we have identified two highly active GH20 6S-GlcNAcases: the WG enzyme from *Prevotella sp.* and the YG enzyme from *Alloprevotella* sp. We then established the sulfate-binding motifs for both enzymes through rigorous biochemical characterization, including site-directed mutagenesis and experimental activity-based screening, shedding more light on how the partially solvated sulfate group is recognized by these GHs with specificity toward sulfated sugar substrates. Subsequently, we used Sequence Similarity Networks (SSN) to further expand the pool of sulfoglycosidases. This approach provided a robust method for discovering catalytically important residues within the substrate-binding pockets.

## Materials and Methods

### Plasmids

Plasmids pJS119K–F3–ORF26(22–773)-6His encoding the F3-ORF26 gene were kindly provided by the Léa Chuzel^18^ from New England BioLabs. Plasmids for all variants were generated by SDM from pJS119 K-ORF26(22–773)-6His using the PrimeSTAR Max kit with primers described in SI Table 1.

Plasmids pET23-Bt4394(22–546)-6His encoding the Bt4394_WT_ gene were kindly provided by the He Lab. Plasmids for all variants were generated by SDM using a PrimeSTAR Max kit with primers described in SI Table 1.

The gene fragments for WG (Uniport: R6ARV4, GenBank: CDA43927.1) were synthesized by GeneArt of Thermo Fisher Scientific, which were optimized for gene expression in *E. coli* and amplified using PrimeSTAR Max DNA Polymerase with primer_WG-F1-f, primer_WG-F1-r, primer_WG-F2-f, and primer_WG-F2-r. The pET23 backbone was amplified using PrimeSTAR Max DNA Polymerase with primer_pET23-WGbackbone-f and primer_pET23-WGbackbone-r. The PCR product of fragments was then assembled into the pET23 vector backbone by using the Gibson Assembly to yield pET23-WG(22–1284)-6His. Plasmids for all variants were generated by SDM from pET23-WG(22–1284)-6His using PrimeSTAR Max kit with primers described in SI Table 1.

The gene fragments for YG (Uniport: L1MPC2, GenBank: EKX92880.1) were purchased from Thermo Fisher Scientific and amplified using PrimeSTAR Max DNA Polymerase with primer_YG-F1-f, primer_YG-F1-r, primer_YG-F2-f, primer_YG-F2-r, YG-GH20b-f, and YG-GH20b-r. The pET23 backbone was amplified using PrimeSTAR Max DNA Polymerase with primer_pET23-YGbackbone-f and primer_pET23-YGbackbone-r. The PCR product of fragments was then assembled into the pET23 vector backbone using the Gibson Assembly to yield pET23-YG (1–1232)-6His. Plasmids for all variants were generated by SDM from pET23-YG (1–1232)-6His using a PrimeSTAR Max kit with primers described in SI Table 1.

### Gibson Assembly

All custom oligonucleotides were purchased from Merck Sigma-Aldrich and listed in Supplementary Table 1. The NEBuilder HiFi DNA assembly master mix was purchased from New England Biolabs. All of the reactions were carried out according to the protocol. Reactions were set up on ice, and then samples were incubated in a thermocycler at 50 °C for 15 min. Samples were stored on ice or at −20 °C for subsequent transformation.

### Site-Directed Mutagenesis

Oligonucleotide primers listed in Supplementary Table 1 were used to carry out site-directed mutagenesis. For all 6-sulfo-β-GlcNAcases, 25 μL PCR reactions were setup in a mixture containing both 5 pmol of forward and reverse primers, 75 ng of template plasmid DNA, and 12.5 μL of PrimeSTAR Max DNA Polymerase Premix (Takara-bio INC). Three steps were used for all the reactions as follows: 98 °C for 10 s, 55 °C for 5 s, 72 °C for 5 s/kb for 35 cycles, and finally held at 4 °C. PCR products were immediately digested by FastDigest DpnI (Thermo Fisher Scientific) for 40 min at 37 °C and transformed into *E. coli* XL1-Blue competent cells. The plasmids were purified using a miniprep kit (QIAGEN, Germany) and subsequently verified by sequencing to ensure that mutations were successful.

### Methods for Gene Expression and Protein Purification

#### F3-ORF26 Sulfoglycosidase

The genes of wild-type F3-ORF26 and variants were expressed in the NEBExpress *I*^*q*^*E. coli* strain (NEB). The transformed cells were incubated on LB agar with 50 μg/mL kanamycin overnight at 37 °C. Transformed cells were grown in LB media containing 50 μg/mL kanamycin at 37 °C until the OD_600_ reached 0.4–0.6. The culture was supplemented with isopropyl-β-d-thiogalactopyranoside (IPTG) to a final concentration of 0.4 mM and then further incubated for 4 h at 37 °C and 200 rpm. Cells were subsequently harvested by centrifugation at 4500 rpm for 25 min at 4 °C, resuspended in buffer A (Tris-HCl 20 mM, pH 7.0, imidazole 50 mM, NaCl 300 mM, Protease Inhibitor Cocktail (half tablet, Sigma-Aldrich)), and incubated at 4 °C for 30 min. The cells were lysed by sonication, and the lysate was centrifuged at 20000 rpm and 4 °C for 25 min. The supernatant was filtered through a 0.45 μm syringe filter before being loaded onto a pre-equilibrated 5 mL HP HisTrap column (GE Healthcare). Subsequently, the column was washed with Buffer A until UV absorbance decreased to baseline, and the protein was eluted with 10% ∼ 80% gradient Buffer B (Tris-HCl 20 mM, pH 7.0, imidazole 500 mM, NaCl 300 mM, Protease Inhibitor Cocktail (half tablet, Sigma-Aldrich)). The purity of the fractions was assessed by SDS-PAGE and the fractions containing F3-ORF26 protein were combined and concentrated before being purified further on size exclusion chromatography (SEC, GE Superdex75 26/600) in buffer C (Tris-HCl 20 mM, pH 7.0, NaCl 300 mM, Protease Inhibitor Cocktail (half tablet, Sigma-Aldrich)).

#### Bt4394

The genes of all Bt4394 wild-type and variants were expressed in the *E. coli* BL21 (DE3) strain (Sigma-Aldrich). The transformed cells were selected with 100 μg/mL ampicillin on LB agar by overnight incubation at 37 °C. Transformed cells were grown in 1 L of LB media containing 100 μg/mL ampicillin at 37 °C until the OD600 reached 0.6. The culture was cooled to 18 °C, supplemented with 0.5 mM IPTG, and then incubated for 20 h at 180–200 rpm.

#### WG and YG Sulfoglycosidases

The genes of wild-type WG and YG sulfoglycosidases and their variants were expressed in the BL21(DE3) Star *E. coli* strain (Sigma-Aldrich). The transformed cells were incubated on LB agar with 100 μg/mL ampicillin overnight at 37 °C. Transformed cells were grown in LB media containing 100 μg/mL ampicillin at 37 °C until the OD600 reached 0.6. The culture was cooled to 20–25 °C before a final concentration of 0.8 mM IPTG was added followed by further incubation for 16 h at 200 rpm.

For Bt4394, WG, and YG sulfoglycosidases, cells were subsequently harvested by centrifugation at 4500 rpm for 25 min at 4 °C, resuspended in buffer D (Tris-HCl 20 mM, pH 8.0, imidazole 50 mM, NaCl 300 mM, PMSF 1 mM), and incubated at 4 °C for 30 min. The cells were lysed by sonication, and the lysate was centrifuged at 10000 rpm, 4 °C for 30 min. The supernatant was filtered through a 0.45 μm syringe filter before being loaded onto a buffer B pre-equilibrated 5 mL HisTrap FF column (GE Healthcare). Subsequently, the column was washed with buffer D until the UV absorbance decreased to baseline, and the protein was eluted with 10% ∼ 80% gradient Buffer E (Tris-HCl 20 mM, pH 8.0, NaCl 300 mM, imidazole 500 mM). The fractions containing the eluted protein, confirmed by SDS-PAGE, were concentrated and further purified by SEC on a GE Superdex75 or Superdex200 26/600 column with buffer F (Tris-HCl 20 mM, pH 8.0, NaCl 300 mM).

For all proteins, the purity of the fractions from SEC was assessed by SDS-PAGE and all those that were >95% pure were combined and concentrated before the kinetics measurements and crystallization screening.

### pH Profile of Sulfoglycosidase Activities

All the initial rates of reactions were performed by monitoring the fluorescence change for 1 min at 25 °C. 18.6 mM 4MU-6S-GlcNAc (Merck) (18.6 mM) dissolved in deionized water was used as substrate stock before being diluted into the respective reaction buffers. All wild-type F3-ORF26, WG, and YG sulfoglycosidases were assayed by measuring the fluorescence of the released 4-methylumbelliferone (4MU) at λ_ex_ = 360 ± 10 nm and λ_em_ = 450 ± 10 nm. The buffer used contained 25 mM Bis-tris propane, 25 mM citrate, and 300 mM NaCl and was titrated with HCl to the final full range of pHs. All experiments were performed in triplicate.

### Michaelis–Menten Kinetics

Michaelis–Menten kinetics for wild-type 6-sulfo-β-GlcNAcases, F3-ORF26, WG and YG, and their variants were measured and compared for the enzyme-catalyzed hydrolysis of 4MU-6S-GlcNAc (Merck) and 4MU-GlcNAc (Merck). Initial release rates of fluorescent 4MU in 100 μL reactions were monitored continuously at λ_ex_ = 360 nm and λ_em_ = 450 nm using a BMG Fluostar microplate reader. All reactions were performed at 25 °C in a buffer containing 25 mM Bis-tris propane, 25 mM citrate, and 300 mM NaCl and titrated with HCl to pH 6.0, except for Bt4394 variants all kinetics were measured at optimal pH 5.5. The concentration of the 4MU formed was assessed using a 4MU standard curve in the same buffer as the kinetics assays. Kinetic parameters (*k*_cat_, *K*_M_, *k*_cat_/*K*_M_) were calculated using the Michaelis–Menten equation *y* = *E*_t_ × *k*_cat_ × *x*/(*K*_M_ + *x*), as in the GraphPad Prism 6.01 Software. All experiments were performed in triplicate.

### Bioinformatics

Sequence Similarity Networks (SSNs), generated using the EFI web tools, are designed to aid in the assignment of in vitro enzymatic activities by exploring the sequence-function space within enzyme families. Essentially, SSNs are networks that illustrate pairwise sequence relationships among groups of homologous proteins. Each protein is depicted as a “node”, and pairs of nodes are connected by an ‘edge’ if they share a pairwise sequence similarity (measured by an alignment score derived from the BLAST bit score) that surpasses a user-defined threshold, the alignment score threshold (AST). By incrementally raising the alignment score threshold for removing edges, the nodes can be segregated into clusters that define isofunctional families. These SSNs are analyzed using Cytoscape, an open-source software platform for visualizing complex networks.^36^

All SSNs were generated using the EFI tools. The SSN for the protein family PF00728 was generated using the “domain” option while excluding fragments using database version UniProt 2024–04^37^ and InterPro 101.^38^ For the SSN identifying WG and YG enzymes, the alignment score threshold (AST) was set to 130, based on our previous study.^15^ In contrast to our previously used UniRef90 SSN, this SSN was generated at full resolution with the most recent version of the databases. The SSN obtained was submitted to the EFI’s color SSN utility, and the domain-delimited FASTA sequences obtained were downloaded for further analysis. A Python script was developed to identify sequences containing the exact motif ‘YYINR’. Of the 43,600 sequences analyzed, 86 (0.20%) contained this exact pattern. The identified sequences were then mapped to the generated SSN. MSAs were generated using EMBL-EBI Clustal Omega implementation^39^ and visualized in Jalview.^40^ Sequences in which the motif YYINR was not aligned with that of Bt4394 were excluded as they were unlikely to be involved in substrate binding, and their presence could be due to random chance, given the large number of sequences. For the focused SSN identifying other 6-sulfo-β-GlcNAcases with the same sulfate-binding motif as F3-ORF26, the GH20 domain sequence of F3-ORF26 was submitted for BLAST against the Uniprot database. To separate the cluster containing F3-ORF26-type 6-sulfo-β-GlcNAcases from other neighboring clusters, a stepwise increase in the AST value from 178 to 202 and finally to 250 was used.

### AlphaFold Structure Prediction

The structures of F3-ORF26, WG, and YG 6-sulfo-β-GlcNAcases were predicted independently using AlphaFold 2 and 3 web servers. The protein sequences were loaded onto the webpage with the default setting for structure predictions, and the prediction results were returned generally after several minutes. Only structures that had a confidence score (pLDDT) above 90 were used. The resulting protein structures were visualized and analyzed by using PyMOL for structural features and potential functional sites. Structures predicted by AlphaFold 2 were generated by Google Colab (https://colab.research.google.com/github/sokrypton/ColabFold/blob/main/AlphaFold2.ipynb). AlphaFold 3-predicted structures were generated by AlphaFold Server (https://alphafoldserver.com/).

## Supporting Information Available

Protein sequences, kinetics, expression gels, and SSN data supplied as Supporting Information (PDF)

## References

[ref1] Muthana S. M.; Campbell C. T.; Gildersleeve J. C. Modifications of glycans: biological significance and therapeutic opportunities. ACS Chem. Biol. 2012, 7 (1), 31–43. 10.1021/cb2004466. 22195988 PMC3262866

[ref2] Whitelock J. M.; Iozzo R. V. Heparan sulfate: a complex polymer charged with biological activity. Chem. Rev. 2005, 105 (7), 2745–2764. 10.1021/cr010213m. 16011323

[ref3] She Y.-M.; Li X.; Cyr T. D. Remarkable Structural Diversity of N-Glycan Sulfation on Influenza Vaccines. Anal. Chem. 2019, 91 (8), 5083–5090. 10.1021/acs.analchem.8b05372. 30908021

[ref4] Xia B.; Royall J. A.; Damera G.; Sachdev G. P.; Cummings R. D. Altered O-glycosylation and sulfation of airway mucins associated with cystic fibrosis. Glycobiology 2005, 15 (8), 747–775. 10.1093/glycob/cwi061. 15994837

[ref5] Lo-Guidice J. M.; Wieruszeski J. M.; Lemoine J.; Verbert A.; Roussel P.; Lamblin G. Sialylation and sulfation of the carbohydrate chains in respiratory mucins from a patient with cystic fibrosis. J. Biol. Chem. 1994, 269 (29), 18794–18813. 10.1016/S0021-9258(17)32238-X. 8034632

[ref6] Kenny D.; Hayes C. A.; Jin C.; Karlsson N. G. Perspective and Review of Mass Spectrometric Based Sulfoglycomics of N-Linked and O-Linked Oligosaccharides. Curr. Proteomics 2011, 8 (4), 278–296. 10.2174/157016411798220853.

[ref7] Carlson T. L.; Lock J. Y.; Carrier R. L. Engineering the Mucus Barrier. Annu. Rev. Biomed. Eng. 2018, 20, 197–220. 10.1146/annurev-bioeng-062117-121156. 29865871 PMC6463277

[ref8] Boltin D.; Perets T. T.; Vilkin A.; Niv Y. Mucin function in inflammatory bowel disease: an update. J. Clin. Gastroenterol. 2013, 47 (2), 106–111. 10.1097/MCG.0b013e3182688e73. 23164684

[ref9] Tsai H. H.; Sunderland D.; Gibson G. R.; Hart C. A.; Rhodes J. M. A novel mucin sulphatase from human faeces: its identification, purification and characterization. Clin. Sci. 1992, 82 (4), 447–454. 10.1042/cs0820447. 1315656

[ref10] Robinson C. V.; Elkins M. R.; Bialkowski K. M.; Thornton D. J.; Kertesz M. A. Desulfurization of mucin by Pseudomonas aeruginosa: influence of sulfate in the lungs of cystic fibrosis patients. J. Med. Microbiol. 2012, 61 (Pt 12), 1644–1653. 10.1099/jmm.0.047167-0. 22918866

[ref11] Larsson J. M. H.; Thomsson K. A.; Rodríguez-Piñeiro A. M.; Karlsson H.; Hansson G. C. Studies of mucus in mouse stomach, small intestine, and colon. III. Gastrointestinal Muc5ac and Muc2 mucin O-glycan patterns reveal a regiospecific distribution. Am. J. Physiol.: Gastrointest. Liver Physiol. 2013, 305 (5), G357–G363. 10.1152/ajpgi.00048.2013. 23832516 PMC3761246

[ref12] Byrd-Leotis L.; Lasanajak Y.; Bowen T.; Baker K.; Song X.; Suthar M. S.; Cummings R. D.; Steinhauer D. A. SARS-CoV-2 and other coronaviruses bind to phosphorylated glycans from the human lung. Virology 2021, 562, 142–148. 10.1016/j.virol.2021.07.012. 34325286 PMC8299723

[ref13] Luis A. S.; Jin C.; Pereira G. V.; Glowacki R. W. P.; Gugel S. R.; Singh S.; Byrne D. P.; Pudlo N. A.; London J. A.; Basle A.; Reihill M.; Oscarson S.; Eyers P. A.; Czjzek M.; Michel G.; Barbeyron T.; Yates E. A.; Hansson G. C.; Karlsson N. G.; Cartmell A.; Martens E. C. A single sulfatase is required to access colonic mucin by a gut bacterium. Nature 2021, 598 (7880), 332–337. 10.1038/s41586-021-03967-5. 34616040 PMC9128668

[ref14] Ghosh D. Human sulfatases: a structural perspective to catalysis. Cell. Mol. Life. Sci. 2007, 64 (15), 2013–2022. 10.1007/s00018-007-7175-y. 17558559 PMC11136368

[ref15] Zhang Z.; Dong M.; Zallot R.; Blackburn G. M.; Wang N.; Wang C.; Chen L.; Baumann P.; Wu Z.; Wang Z.; Fan H.; Roth C.; Jin Y.; He Y. Mechanistic and Structural Insights into the Specificity and Biological Functions of Bacterial Sulfoglycosidases. ACS Catal. 2023, 13 (1), 824–836. 10.1021/acscatal.2c05405.

[ref16] Katoh T.; Maeshibu T.; Kikkawa K.-i.; Gotoh A.; Tomabechi Y.; Nakamura M.; Liao W.-H.; Yamaguchi M.; Ashida H.; Yamamoto K.; Katayama T. Identification and characterization of a sulfoglycosidase from Bifidobacterium bifidum implicated in mucin glycan utilization. Biosci., Biotechnol., Biochem. 2017, 81 (10), 2018–2027. 10.1080/09168451.2017.1361810. 28814130

[ref17] Rho J.-h.; Wright D. P.; Christie D. L.; Clinch K.; Furneaux R. H.; Roberton A. M. A Novel Mechanism for Desulfation of Mucin: Identification and Cloning of a Mucin-Desulfating Glycosidase (Sulfoglycosidase) from Prevotella Strain RS2. J. Bacteriol. 2005, 187 (5), 1543–1551. 10.1128/JB.187.5.1543-1551.2005. 15716424 PMC1064001

[ref18] Chuzel L.; Fossa S. L.; Boisvert M. L.; Cajic S.; Hennig R.; Ganatra M. B.; Reichl U.; Rapp E.; Taron C. H. Combining functional metagenomics and glycoanalytics to identify enzymes that facilitate structural characterization of sulfated N-glycans. Microb. Cell Fact. 2021, 20 (1), 162 10.1186/s12934-021-01652-w. 34419057 PMC8379841

[ref19] Bains R. K.; Nasseri S. A.; Liu F.; Wardman J. F.; Rahfeld P.; Withers S. G. Characterization of a New Family of 6-Sulfo-N-Acetylglucosaminidases. J. Biol. Chem. 2023, 299 (10), 105214 10.1016/j.jbc.2023.105214. 37660924 PMC10570127

[ref20] Zeng X.; Sun Y.; Ye H.; Liu J.; Uzawa H. Synthesis of p-nitrophenyl sulfated disaccharides with β-d-(6-sulfo)-GlcNAc units using β-N-acetylhexosaminidase from Aspergillus oryzae in a transglycosylation reaction. Biotechnol. Lett. 2007, 29 (7), 1105–1110. 10.1007/s10529-007-9366-x. 17492477

[ref21] Uzawa H.; Zeng X.; Minoura N. Synthesis of 6′-sulfodisaccharides by β-N-acetylhexosaminidase-catalyzed transglycosylation. Chem. Commun. 2003, 1, 100–101. 10.1039/B209893H. 12610985

[ref22] Tegl G.; Hanson J.; Chen H. M.; Kwan D. H.; Santana A. G.; Withers S. G. Facile Formation of β-thioGlcNAc Linkages to Thiol-Containing Sugars, Peptides, and Proteins using a Mutant GH20 Hexosaminidase. Angew. Chem., Int. Ed. Engl. 2019, 58 (6), 1632–1637. 10.1002/anie.201809928. 30549167 PMC6637381

[ref23] Kobata A. Exo- and endoglycosidases revisited. Proc. Jpn. Acad. B: Phys. Biol. Sci. 2013, 89 (3), 97–117. 10.2183/pjab.89.97. PMC3647078 23474886

[ref24] Rudd P. M.; Dwek R. A. Rapid, sensitive sequencing of oligosaccharides from glycoproteins. Curr. Opin. Biotechnol. 1997, 8 (4), 488–497. 10.1016/S0958-1669(97)80073-0. 9265730

[ref25] Kresse H.; Fuchs W.; Glössl J.; Holtfrerich D.; Gilberg W. Liberation of N-acetylglucosamine-6-sulfate by human beta-N-acetylhexosaminidase A. J. Biol. Chem. 1981, 256 (24), 12926–12932. 10.1016/S0021-9258(18)42985-7. 6458607

[ref26] Jumper J.; Evans R.; Pritzel A.; Green T.; Figurnov M.; Ronneberger O.; Tunyasuvunakool K.; Bates R.; Žídek A.; Potapenko A.; Bridgland A.; Meyer C.; Kohl S. A. A.; Ballard A. J.; Cowie A.; Romera-Paredes B.; Nikolov S.; Jain R.; Adler J.; Back T.; Petersen S.; Reiman D.; Clancy E.; Zielinski M.; Steinegger M.; Pacholska M.; Berghammer T.; Bodenstein S.; Silver D.; Vinyals O.; Senior A. W.; Kavukcuoglu K.; Kohli P.; Hassabis D. Highly accurate protein structure prediction with AlphaFold. Nature 2021, 596 (7873), 583–589. 10.1038/s41586-021-03819-2. 34265844 PMC8371605

[ref27] Zallot R.; Oberg N. O.; Gerlt J. A. ‘Democratized’ genomic enzymology web tools for functional assignment. Curr. Opin. Chem. Biol. 2018, 47, 77–85. 10.1016/j.cbpa.2018.09.009. 30268904 PMC6289791

[ref28] Zallot R.; Oberg N.; Gerlt J. A. The EFI Web Resource for Genomic Enzymology Tools: Leveraging Protein, Genome, and Metagenome Databases to Discover Novel Enzymes and Metabolic Pathways. Biochemistry 2019, 58 (41), 4169–4182. 10.1021/acs.biochem.9b00735. 31553576 PMC7057060

[ref29] Oberg N.; Zallot R.; Gerlt J. A. EFI-EST, EFI-GNT, and EFI-CGFP: Enzyme Function Initiative (EFI) Web Resource for Genomic Enzymology Tools. J. Mol. Biol. 2023, 435 (14), 168018 10.1016/j.jmb.2023.168018. 37356897 PMC10291204

[ref30] Bowman K. G.; Bertozzi C. R. Carbohydrate sulfotransferases: mediators of extracellular communication. Chem. Biol. 1999, 6 (1), R9–R22. 10.1016/S1074-5521(99)80014-3. 9889154

[ref31] Hemmerich S. Carbohydrate sulfotransferases: novel therapeutic targets for inflammation, viral infection and cancer. Drug Discovery Today 2001, 6 (1), 27–35. 10.1016/S1359-6446(00)01581-6. 11165170

[ref32] Simanek E. E.; McGarvey G. J.; Jablonowski J. A.; Wong C. H. Selectin-Carbohydrate Interactions: From Natural Ligands to Designed Mimics. Chem. Rev. 1998, 98 (2), 833–862. 10.1021/cr940226i. 11848916

[ref33] Terwilliger T. C.; Liebschner D.; Croll T. I.; Williams C. J.; McCoy A. J.; Poon B. K.; Afonine P. V.; Oeffner R. D.; Richardson J. S.; Read R. J.; Adams P. D. AlphaFold predictions are valuable hypotheses and accelerate but do not replace experimental structure determination. Nat. Methods 2024, 21 (1), 110–116. 10.1038/s41592-023-02087-4. 38036854 PMC10776388

[ref34] Katoh T.; Yamada C.; Wallace M. D.; Yoshida A.; Gotoh A.; Arai M.; Maeshibu T.; Kashima T.; Hagenbeek A.; Ojima M. N.; Takada H.; Sakanaka M.; Shimizu H.; Nishiyama K.; Ashida H.; Hirose J.; Suarez-Diez M.; Nishiyama M.; Kimura I.; Stubbs K. A.; Fushinobu S.; Katayama T. A bacterial sulfoglycosidase highlights mucin O-glycan breakdown in the gut ecosystem. Nat. Chem. Biol. 2023, 19 (6), 778–789. 10.1038/s41589-023-01272-y. 36864192

[ref35] Abramson J.; Adler J.; Dunger J.; Evans R.; Green T.; Pritzel A.; Ronneberger O.; Willmore L.; Ballard A. J.; Bambrick J.; Bodenstein S. W.; Evans D. A.; Hung C.-C.; O’Neill M.; Reiman D.; Tunyasuvunakool K.; Wu Z.; Žemgulytė A.; Arvaniti E.; Beattie C.; Bertolli O.; Bridgland A.; Cherepanov A.; Congreve M.; Cowen-Rivers A. I.; Cowie A.; Figurnov M.; Fuchs F. B.; Gladman H.; Jain R.; Khan Y. A.; Low C. M. R.; Perlin K.; Potapenko A.; Savy P.; Singh S.; Stecula A.; Thillaisundaram A.; Tong C.; Yakneen S.; Zhong E. D.; Zielinski M.; Žídek A.; Bapst V.; Kohli P.; Jaderberg M.; Hassabis D.; Jumper J. M. Accurate structure prediction of biomolecular interactions with AlphaFold 3. Nature 2024, 630 (8016), 493–500. 10.1038/s41586-024-07487-w. 38718835 PMC11168924

[ref36] Shannon P.; Markiel A.; Ozier O.; Baliga N. S.; Wang J. T.; Ramage D.; Amin N.; Schwikowski B.; Ideker T. Cytoscape: a software environment for integrated models of biomolecular interaction networks. Genome Res. 2003, 13 (11), 2498–2504. 10.1101/gr.1239303. 14597658 PMC403769

[ref37] UniProt: the universal protein knowledgebase in 2021. Nucleic Acids Res. 2021, 49 (D1), D480–D489. 10.1093/nar/gkaa1100. 33237286 PMC7778908

[ref38] Paysan-Lafosse T.; Blum M.; Chuguransky S.; Grego T.; Pinto B. L.; Salazar G. A.; Bileschi M. L.; Bork P.; Bridge A.; Colwell L.; Gough J.; Haft D. H.; Letunić I.; Marchler-Bauer A.; Mi H.; Natale D. A.; Orengo C. A.; Pandurangan A. P.; Rivoire C.; Sigrist C. J. A.; Sillitoe I.; Thanki N.; Thomas P. D.; Tosatto S. C. E.; Wu C. H.; Bateman A. InterPro in 2022. Nucleic Acids Res. 2023, 51 (D1), D418–D427. 10.1093/nar/gkac993. 36350672 PMC9825450

[ref39] Madeira F.; Madhusoodanan N.; Lee J.; Eusebi A.; Niewielska A.; Tivey A. R. N.; Lopez R.; Butcher S. The EMBL-EBI Job Dispatcher sequence analysis tools framework in 2024. Nucleic Acids Res. 2024, 52 (W1), W521–W525. 10.1093/nar/gkae241. 38597606 PMC11223882

[ref40] Waterhouse A. M.; Procter J. B.; Martin D. M.; Clamp M.; Barton G. J. Jalview Version 2 – a multiple sequence alignment editor and analysis workbench. Bioinform. 2009, 25 (9), 1189–1191. 10.1093/bioinformatics/btp033. PMC2672624 19151095

