# Supplementary material for: Bioinformatics-Facilitated Identification of Novel Bacterial Sulfoglycosidases That Hydrolyze 6-Sulfo-N-acetylglucosamine
Source: ACS Bio Med Chem Au. 2024 Nov 19;4(6):342–52. doi: 10.1021/acsbiomedchemau.4c00088 (PMC11659886; doi:10.1021/acsbiomedchemau.4c00088)
Supplement: Supplementary file 1 — bg4c00088_si_001.pdf [file bg4c00088_si_001.pdf]

Supporting Information for

Bioinformatics-Facilitated Identification of Novel Bacterial Sulfoglycosidases that Hydrolyze 6-Sulfo-*N*-acetylglucosamine

Mochen Dong[+], Zhuoyun Chen[&∇], Yuan He[⊥], Rémi Zallot[^], Yi Jin[&∇*]

[+] School of Chemistry, Cardiff University, Cardiff, CF10 3AT, United Kingdom

[⊥] Key Laboratory of Synthetic and Natural Functional Molecule, College of Chemistry and Materials Science, Northwest University, Xi'an, 710127, P. R. China

[^] Department of Life Sciences, Manchester Metropolitan University, Dalton Building, Chester Street, Manchester, M1 5GD, United Kingdom

[&] Manchester Institute of Biotechnology, University of Manchester, 131 Princess Street, Manchester, M1 7DN, United Kingdom

[∇] Department of Chemistry, School of Natural Sciences, Faculty of Science and Engineering, University of Manchester, Oxford Road, Manchester, M13 9PL, United Kingdom

Corresponding Author

Yi Jin – orcid.org/0000-0002-6927-4371; Email: yi.jin@manchester.ac.uk, Phone: +44(0)1615294338

List of Figures

Supplementary Figure 1. The standard curves of 4MU in the universal buffer at various pHs. Curves **a** to **q** fitted by linear regression. 6

Supplementary Figure 2. Michaelis-Menten plots for hydrolysis of 4MU-6S-GlcNAc by F3-ORF26 variants at pH 6.0. 7

Supplementary Figure 3. F3-ORF26 6-sulfo-β-GlcNAcases (highlighted as a yellow dot) mapped on the focused Uniport SSN created for the GH20 domain. 9

Supplementary Figure 4 (**a**) Multiple sequence alignment (MSA) and (**b**) sequence logo highlighting the conserved sulfate-binding feature in the F3-ORF26 protein cluster (cluster 2 in blue in Supplementary Figure 8c). 13

Supplementary Figure 5. Full-resolution Sequence Similarity Network (SSN) for the GH20 Domain with an Alignment Score Threshold (AST) of 130 (PF00728, Domain, no fragments). 14

Supplementary Figure 6. The WG_{WT} and YG_{WT} structure predicted by AlphaFold 2, and its GH20 catalytic domain aligned with the $Bt4394_{D335N}$-6S-NAG-oxazoline intermediate (PDB: 7DVB). The 6S-NAG-oxazoline ligand from PDB:7DVB is in green. H-bond distances are for donor-to-acceptor in Å. 15

Supplementary Figure 7. Michaelis-Menten plots for hydrolysis of 4MU-6S-GlcNAc by WG variants at pH 6.0. 16

Supplementary Figure 8. Michaelis-Menten plots for hydrolysis of 4MU-6S-GlcNAc by YG variants at pH 6.0. 17

Supplementary Figure 9. Overlay the structures of the 6S-NAG-oxazoline intermediate complexes of $Bt4394_{D335N}$ (PDB: 7DVB, in cyan) in comparison with the $Bt4394_{Q431W,\ I432G}$ generated by (**a**) AlphaFold 3 and (**b**) AlphaFold 2. $Bt4394_{D335N}$ (PDB: 7DVB, in cyan) in comparison with the $Bt4394_{Q431Y,\ I432G}$ structures generated by (**c**) AlphaFold 3 and (**d**) AlphaFold 2 with the default settings. The 6S-NAG-oxazoline ligand from PDB:7DVB is in green. H-bond distances are for donor-to-acceptor in Å. 18

Supplementary Figure 10. Michaelis-Menten plots for illustrating substrate specificity of sulfated substrate 4MU-6S-GlcNAc and non-sulfated 4MU-GlcNAc with $Bt4394_{WT}$ and its variants $Bt4394_{Q431W,I432G}$ and $Bt4394_{Q431Y,\ I432G}$ at pH 5.5. 19

Supplementary Figure 11. SDS-PAGE gels for F3-ORF26 protein purification. 20

Supplementary Figure 12. SDS-PAGE gels for expression and purification of WG protein. 20

Supplementary Figure 13. SDS-PAGE gels for expression and purification of YG protein. 21

Supplementary Figure 14. SDS-PAGE gels for expression test of truncated YG protein. 22

Molecular cloning and protein sequences

Bt4394: dsDNA encoding Bt4394 (GenBank: AAO79499.1 from Bacteroides thetaiotaomicron VPI-5482, Uniprot ID: Q89ZI3) from residue Q22 (without the signal peptide sequence in bold) was codon optimized for *E. coli*, synthesized (Sangon Biotech, Shanghai) and cloned into the pET23(a) expression vector using the *Nde*I/*Xho*I restriction sites. The resulting construct encoded a C-terminal His6-tagged recombinant protein as below.

MQEIALTPQPAHLTVKDGRFEFGNQLKAKVTPYQGDSIRMVFESFKKELQEATGIKVSSTQKEAKARIILDLNPQLPAEAYKL
NVSKKQVRIEASRPAGFYYALQTLKQLMPRNVMAGVATSDHSQWSLPSVEIEDAPRFEWRGFMLDEGRHFFGKDEIKRVIDMM
AIYKMNRFHWHLTEDQGWRIEIKKYPKLTETGAWRNSKVLAYGDVKPDGERYGGFYTQKDIKEIVAYAKKKFIEIIPEIDIPG
HSQAAVAAYPEFLACDPRDKHEVWLQQGISTDVINVANPKAMQFAKEVIDELTELFPFNYIHLGGDECPTRKWQKNDECKKLL
SEIGSSNFRDLQIYFYKQLKDYIATKPADQQRQLIFWNEVLHGNTSILGNDITIMAWIGANAAAKQAAKQGMNTILSPQIPYY
INRKQSKLPTEPMSQGHGTETVEAVYNYQPLKDVDAALQPYYKGVQANFWTEWVTEPSVLEYLMLPRLAAVAEAGWTPQEKRN
YEDFKERIRKDAELYDLKGWNYGKHIMKLEHHHHHH

WG Sulfoglycosidase: dsDNA encoding beta-N-acetylhexosaminidase (GeneBank: CDA43927.1 from *Prevotella sp.* CAG:5226, Uniprot ID: R6ARV4) from residue Q22 (without the signal peptide sequence in bold) was codon optimized for *E.coli*, synthesized by Gene Art (Thermo Fisher Scientific, Germany) and cloned into the pET23(a) expression vector using the *Nde*I/*Xho*I restriction sites. The resulting construct encoded a C-terminal His6-tagged recombinant protein as below.

MQTFVNLTPRPASMTVGTGSLALPSQFTVSYTGLDEDGVNEVNQFAKSYTDVTGAAVSVAADDASALFQVSLLPASSSLKTDG
YKLDITDSKVTIQAKSALGLFYAFQSVKKMLPANVMAGVKDATVTSYPLPVVSITDQPRFDYRGFMLDVSRHFFTANEVKRII
DLMAAYKMNTFHWHLTDDQGWRVPIKKYPKLTTIGATAPNRRYTDMYELTQYWINKPYGPYSYTEEEIKDVVAYAKARHIDIV
PEIDMPGHFSAAMTAYPEFSCTPDGAHNVWDDGGISNDILNVANPKAVQFAKDILEELMELFPGKYIHIGGDECPTYAWEKNA
ECQALYKELKLTHYRQLQSHFIQQLDEFVKSKGRHLAIWNEGITAGGADTDIMKSTGAVVYCWTNPEAAVNQAKKLGMPSIYT
PWGPYYINRRQGNGPTDPPGAGSGSDNVQATYNQTIPSATTYGVQGTFWCEHVSDTDYLEWLALPRLIAVAERGWTPESGKNF
SDFQKRMSADTVMLNYGNYKYCKYFMLDNGQGGGTTMVMPHANTADKKYYYRIISGGTDNTRAGRCIELLADGSSLISANSGK
GAAAGVLWTSPQAAEGDANYNYQWWSIEEDPANPGKYALVCMAQPDGSVKPDPSAVSTAGRWSYDNAAKHYNFVLGSGSYGKK
GDNYYYTIASDKVNGQYLNSSMGGQGLAVNLYSNPNDGNGGCWEFSPKEDYGGGSGTKPVTFDYLEEGKTYMLTNAVEGFESI
TLSDDNNGNSLKANNNAFANNAWTVEASTINADGSQTVKVKNVATGRYINSLGAYADRVGCPVNMGNATADANVTISYVKNYD
DLRIKVGGKSLFAVPSGVANAGATTGAGVSYDAARNQGAEWTATEVKIVTLNCQDDKGVNLGTFKRSVPVSVTEITGDLCPTF
KNTAVEHIEADGDNNYNVTYKRSAYSLNIVCADKAGVLISKDEVTVPVGESYTMKVPEAKYCTLLNSDVADGTLITPDADRTI
NVTYEINAIVGVKAEGEPVKELKSNNKYLLYDATTAAGRAGYRAIRDNNVINRYLSAEGMLPTGVWTLQGSGNKFKVLNEYTG
LYVPQLKNSAATTATRAGGDTFTFATNADGTWNIKSTSGQYWDGLASGDLVGWNGGTGHPIRISTFYAQPMFTVNIVCRDADD
DKVILKQSSNLVVAGTAYPIVIPTIEGYTMQSVTGNETYGGTVEDFVNIVVTYKNNATVGINNVSTDNASAAVRIYDLQGRRV
QRVQQPGLYIVNGKKTLVKLEHHHHHH

YG Sulfoglycosidase: dsDNA encoding beta-N-acetylhexosaminidase (GeneBank: EKX92880.1 from *Alloprevotella sp. oral taxon 473 str. F0040*, Uniprot ID: L1MPC2) from the first residue (no signal peptide) was codon optimized for *E.coli*, synthesized by Gene Art (Thermo Fisher Scientific, Germany) and cloned into the pET23(a) expression vector using the *Nde*I/*Xho*I restriction sites. The resulting construct encoded a C-terminal His6-tagged recombinant protein as below.

MQQFVNLTPVPMQMRVDKGTYRLPAQFTIGGAQLPDSIKAEAQKFVTDFNKSATGATASYSKKPEGAALELVHDKTLYKTLGQ
EGYKLAVTATGIRLESATTTGFFYGLTSLKKMLPACIAAGVKDEKVVTYELPLVQITDKPRFPYRGFMLDVARHFFTVQEVKK
MLDVMAIYKLNKFHFHLTEDQGWRWEVKKYPKLTKVGAVASNTYVTSMEHGAYWTNQQYGPYFYTREDLKEIVAYAAAKHIEV
IPEIDMPGHFSAAMAAYPEFSCNPDGVHRVETWGGVFTDVLNVANPKAVRFVKDILDELMEIFPSKNVHIGGDECPTTAWENN
AECQAMYKKLNLTSYRQLQTHFIAEITDYLKRKGRKISVWNETVTEKGADLQLMKKTGATVYCWVPARKGAEIANSLGLPSIY
TVYGPYYINRAPRKEGWMKTLPGNGSDHLKATYNEQPTDFSHSIGVQGTFWTEHVATPDVMEFLALPRLIAVGEAGWSPQNKK
NFDSFVQRMRADTTMLNYAGYSYDRAYLNENKASTMVYPTVSTAEKEVYYRIVTRGNDKERSGRCIELLSNTSPIVAAEKHNG
AQALRLWTAPQAKEGEAAYDYQQWKLEVDPKNPERFALVSKAYPEGSVAASPTAVAVNGRWNYDTKAKYYDFILGDKGYGVAD
GYRYYTIRSAKHDGQWWNASMSRQGLAVNVYTNPTDGNGGLWSFVGGTPVGEEVPMVEAPAVLPKSGQVYVVRNTVAGHAGAM
LADDGQQQGLIHTLSGQNANNGWEVVNASAFDTKGGTMTMQLRNVTTQRFVGQPETQKVERFGFPVTMSKTPTKITLTYAPKT
TDFTLASGGKFLFPVSASAPQLQGRVSSGSGVGGDNAVRPQGTGWELVPARAVTYRCVTTDGKELQTITEYLPTDATQVTVPQ
FKGYKVKGEIPQLQQGNEAQTFTIVYKKSKHLVFLDAINDDGTLLAQDTIAVPVGESVVIRAPKIDFFSLKEFPEEGIKLTPT
DDVYRTIVYTTSALLGVKALATPLKELKDGQQVLIYDFSTKDPNRAGFRNVSATSGRVLQGGLNNGEASPQFVWKVQKKGSHW
QFFHPATALFMPVLKESKEVIVAKEAGQFSASRNADGTWKVQGTNGQYWDGVVGGMTGWHTYGHPYQFYTFVAAPYFRLTVRY
VDTEGKSVLPSEQSIVPAGSEVTVVAPEIKDFKWKETISMLSDLKRIDAHGDITVVYEKATGIGSVTGAKSFGHVSTYDLQGR
RVEKARHGLFIVNGKKLLTLKVLEHHHHHH

F3-ORF26 Sulfoglycosidase: (GeneBank: GKH82764.1 from *Phocaeicola dorei*, Uniprot ID: A0A4R4I8J5) from residue Q23 (without the signal peptide sequence in bold) was codon optimized for *E.coli*, and cloned into the pJS119K expression vector with a *Tac* promotor between the *Nde*I/*Eco*RI restriction sites. The resulting construct encoded a C-terminal His6-tagged recombinant protein as below. The whole pJS119K-ORF26-6His construct was kindly provided by Léa Chuzel[1] from New England BioLabs®.

MSPTKTEIGNLNVIPQPQEVSQDIQAHPFVINPQTGIVYPEGNEKLQRTAEFLASYIKEATGITVRTTTEAAKKNSIILAVDS
SITNKEGYQLEVTSENIHLNGGSESGVFYGMQTLYKALPLTKNKQVSAAIPVGTVNDYPRFGYRGFMVDVGRHYFPVSYLKQI
IDMLALHNINYFHWHLTEDQGWRIEIKKYPKLTEIGSIRPRTLIDRETQTYDETPHSGFYTQEEAKEIVKYAADRFITVIPEV
DLPGHMMGALVSYPELGCTGGPYEIPCKWGVFPDVLCGGNDRALQFAKDVLNEIMDIFPSPYIHIGGDECPKVRWEKCPACQA
KIRELGLKDTPKHSKENQLQTYFMSEVGKVINDRGRKMLGWDEMLEGGLAPGATVMSWTGVKGGIEAARLHHDAIMTPIQFLY
FSNPTYNRIKGTKSLERVYTFEPVSNELAEDERKYIIGTQGCIWTEWTRDSLKMEWQILPRMAALSEIQWTEPLHKNFDSFLK
RLPALLAIYRDRGYDFRQDIYDVNIDIVPAPDEGKAKIAFQTFDDAEIHYTLDGSVPDVQSPLYTDTIQVDKDVIIQAIAVRP
QGTSQISKEEIHFNAATMRPVTLNTIPHKSYTFKGGSTLIDGLYGDMNYRSGRWIGFYGTDMNVTLDLLEPKEVSSVFVNTML
NTGDAIFGTTGLKVEVSEDGKNFRRVASENFPVVEKGTKMQSRKDSVSFDKVKARYIKIIAEVTPKLPAWHSMPGEKAFLFVD
EIGVEHHHHHH

Table S1. Oligonucleotides used for sequencing and mutagenesis. The letters in bold indicate the sites of mutation. In the primers for Gibson Assembly, lowercase letters indicate overlaps with the backbone vector, while uppercase letters indicate overlaps with the genes.

**Primer**	**Sequence (5' to 3')**
T7	TAATACGACTCACTATAGGG
T7-term	GCTAGTTATTGCTCAGCGG
Bt4394-Q431W, I432G-f	GTATGAACACCATCCTGTCTCCG**TGGGGA**CCGTACTACATCAACCGTAAAC
Bt4394-Q431W, I432G-r	GGAGACAGGATGGTGTTCATACC
Bt4394-Q431Y, I432G-f	GTATGAACACCATCCTGTCTCCG**TATGGA**CCGTACTACATCAACCGTAAAC
Bt4394-Q431Y, I432G-r	GGAGACAGGATGGTGTTCATACC
WG-F1-f	tttaagaaggagatatacaTATGCAGACCTTTGTTAATC
WG-F1-r	TGCACTCGGATCCGGTTTAAC
WG-F2-f	TTAAACCGGATCCGAGTGCAGTTAGCACCGCAGGCCGTTG
WG-F2-r	TTTCACCAGGGTTTTCTTACCATTAAC
pET23-WGbackbone-f	TTTCACCAGGGTTTTCTTACCATTAAC
pET23-WGbackbone-r	ATAtgtatatctccttcttaaagttaaacaaaattatttc

WG-W437F-f	ATGCCGAGCATTTATACCCCG**TTT**GGACCGTATTACATTAATCGTC
WG-W437F-r	GTATAAATGCTCGGCATACCCAGTTTC
WG-W437Q-f	ATGCCGAGCATTTATACCCCG**CAG**GGACCGTATTACATTAATCGTC
WG-W437-r	GTATAAATGCTCGGCATACCCAGTTTC
WG-G438I-f	CGAGCATTTATACCCCGTGG**ATT**CCGTATTACATTAATCGTCGTC
WG-G438I-r	GGGTATAAATGCTCGGCATACCCAGT
WG-N443D-f	CGTGGGGACCGTATTACATT**GAT**CGTCGTCAAGGTAATGGT
WG-N443D-r	AATACGGTCCCCACGGGGTATAAAT
WG-R444A-f	GGGGACCGTATTACATTAATGC**GCG**TCAAGGTAATGGTCCGACC
WG-R444A-r	TGTAATACGGTCCCCACGGGGTATA
YG-F1-f	tttaagaaggagatatacaTATGCCTCGTTTTCCGTATC
YG-F1-r	AACGGCCAGACCTTGACGGCTC
YG-F2-f	TGAGCCGTCAAGGTCTGGCCGTTAATGTTTATACCAATCC
YG-F2-r	ACCGGTTGCTTTTTCATAAAC
GH20b-YG-f	aagaaggagatatacaTATGCAGCAGTTTGTTAATCTGACA
GH20b-YG-r	CGAGGTTTATCGGTAATCTGAACCAGC
pET23-YGbackbone-f	TTTATGAAAAAGCAACCGGTCtcgagcaccaccaccac
pET23-YGbackbone-r	CATAtgtatatctccttcttaaagttaaac
YG-Y439F-f	TGCCGAGCATTTATACCGTT**TTT**GGACCGTATTACATTAATCGTGCAC
YG-Y439A-f	TGCCGAGCATTTATACCGTT**GCG**GGACCGTATTACATTAATCGTGCAC
YG-Y439Q-f	TGCCGAGCATTTATACCGTT**CAG**GGACCGTATTACATTAATCGTGCAC
YG-Y439-r	GGTATAAATGCTCGGCAGACCCAGG
YG-G440I-f	GAGCATTTATACCGTTTAT**ATC**CCGTATTACATTAATCGTGCACC
YG-G440I-r	ACGGTATAAATGCTCGGCAGACCCA
YG-N445D-f	TTTATGGACCGTATTACATT**GAT**CGTGCACCGCGTAAAGATGGT
YG-N445D-r	GTAATACGGTCCATAAACGGTATAA
YG-R446A-f	TATGGACCGTATTACATTAAT**GCG**GCACCGCGTAAAGATGGTTGG
YG-R446A-r	GTAATACGGTCCATAAACGGTATAA
ORF26-Q443E-f	CCATGATGCAATTATGACTCCTATT**GAA**TTTCTTTATTTCAGCAATCCTACTTAC
ORF26-Q443E-r	TAGGAGTCATAATTGCATCATGGTGCAG
ORF26-S438A-f	GACTCCTATTCAGTTTCTTTATTTC**GCG**AATCCTACTTACAATCGGATAAAAG
ORF26-S438A-r	GAAACTGAATAGGAGTCATAATTGCATCATGG
ORF26-N439D-f	CCTATTCAGTTTCTTTATTTCAGC**GAT**CCTACTTACAATCGGATAAAAGG
ORF26-N439D-r	GCTGAAATAAAGAAACTGAATAGGAGTC
ORF26-Y442F-f	CAGTTTCTTTATTTCAGCAATCCTACT**TTT**AATCGGATAAAAGGGACTAAAAGCC
ORF26-Y442F-r	GCTGAAATAAAGAAACTGAATAGGAGTC
ORF26-N443D-f	CTTTATTTCAGCAATCCTACTTAC**GAT**CGGATAAAAGGGACTAAAAGCC
ORF26-N443D-r	GTAGGATTGCTGAAATAAAGAAACTGAATAGG
ORF26-R444A-f	TCAGCAATCCTACTTACAAT**GCG**ATAAAAGGGACTAAAAGCC
ORF26-R444A-r	GTAAGTAGGATTGCTGAAATAAAG
ORF26-SEQ-f	ATGCCGCTGACCGTTTTATTACTGTAATTC
ORF26-SEQ-r	GTTCACATCATAAATATCCTGACGAAAATC

Expression test for truncated YG protein (aa 166-515)

Several constructs were built to improve the expression of truncated YG protein containing GH20 catalytic domain from amino acids 166 to 515. The YG (166-515) gene fragment was amplified using PrimeSTAR Max DNA Polymerase with a pair of primers, which was then assembled into the pET23 vector backbone using Gibson Assembly to yield pET23-YG(166-515)-6His. The pGEX-2T vector backbone was amplified from pGEX2T-hGlcNAckinase using PrimeSTAR Max DNA Polymerase with primer_ GST-backbone-f and primer_ GST-backbone-r.

pGEX2T-YG(166-515)-6His was generated by deletion mutagenesis from pGEX2T-YG (full-length)-6His using PrimeSTAR Max with primers. The pET28a vector containing trigger factor (TF) and ubiquitin (Ub) was already present in the lab. Subsequently, the YG gene fragment was assembled into the vector to yield pET28a-TF-YG(full-length)-6His. pET28a-TF-YG(166-515)-6His was generated by deletion mutagenesis from pET28a-TF-YG(full-length)-6His using PrimeSTAR Max with primers.

The pET23-YG(166-515)-6His plasmids were expressed in *E. coli* BL21(DE3) Star strain and BL21(DE3)pLysS strain (Sigma-Aldrich). pET28a-TF-YG(166-515)-6His plasmids were expressed in BL21(DE3)pLysS strain. The transformed cells were selected with specific antibiotics on Luria-Bertani (LB) agar solid medium by overnight incubation at 37 °C. Transformed cells were grown in 100 mL of LB media containing antibiotics at 37 °C until the OD600 reached 0.6. The culture was cooled to 18 °C before a final concentration of 0.5 mM isopropyl-β-D-thiogalactopyranoside (IPTG, Sigma-Aldrich) was added, and then further incubated for 20 h at 180 rpm. A small fraction of 1 mL culture was harvested by

centrifugation at 13000 rpm for 1 min at 4 °C. Then 60 µL of bugbuster protein extract reagent was added into the wet cell paste followed by 20 min incubation of the cell suspension at room temperature. Collect a sample of cell suspension for SDS-PAGE and remove insoluble cell debris by centrifugation at 13000 rpm for 5 min at 4 °C. Transfer the supernatant to a fresh tube and collect a sample for SDS-PAGE. Load samples including IPTG (-) fraction and BugBuster lysed fractions on SDS-PAGE gels to check the results for expression tests.

Table S2. Equations for 4MU fluorescence standard curves in CBTP buffer

pH	Equation
2.0	Y = 7.496*X + 1.753
2.5	Y = 7.896*X + 2.095
3.0	Y = 9.049*X + 3.037
3.5	Y = 10.17*X + 1.367
4.0	Y = 10.67*X + 3.741
4.5	Y = 10.66*X + 2.255
5.0	Y = 11.63*X + 1.060
5.5	Y = 12.82*X + 3.772
6.0	Y = 16.75*X + 4.661
6.5	Y = 38.25*X + 2.188
7.0	Y = 72.49*X + 0.3575
7.5	Y = 158.2*X - 2.430
8.0	Y = 357.0*X - 12.03
8.5	Y = 480.2*X - 17.45
9.0	Y = 558.8*X + 40.79
9.5	Y = 550.4*X + 21.92
10.0	Y = 481.1*X + 27.43

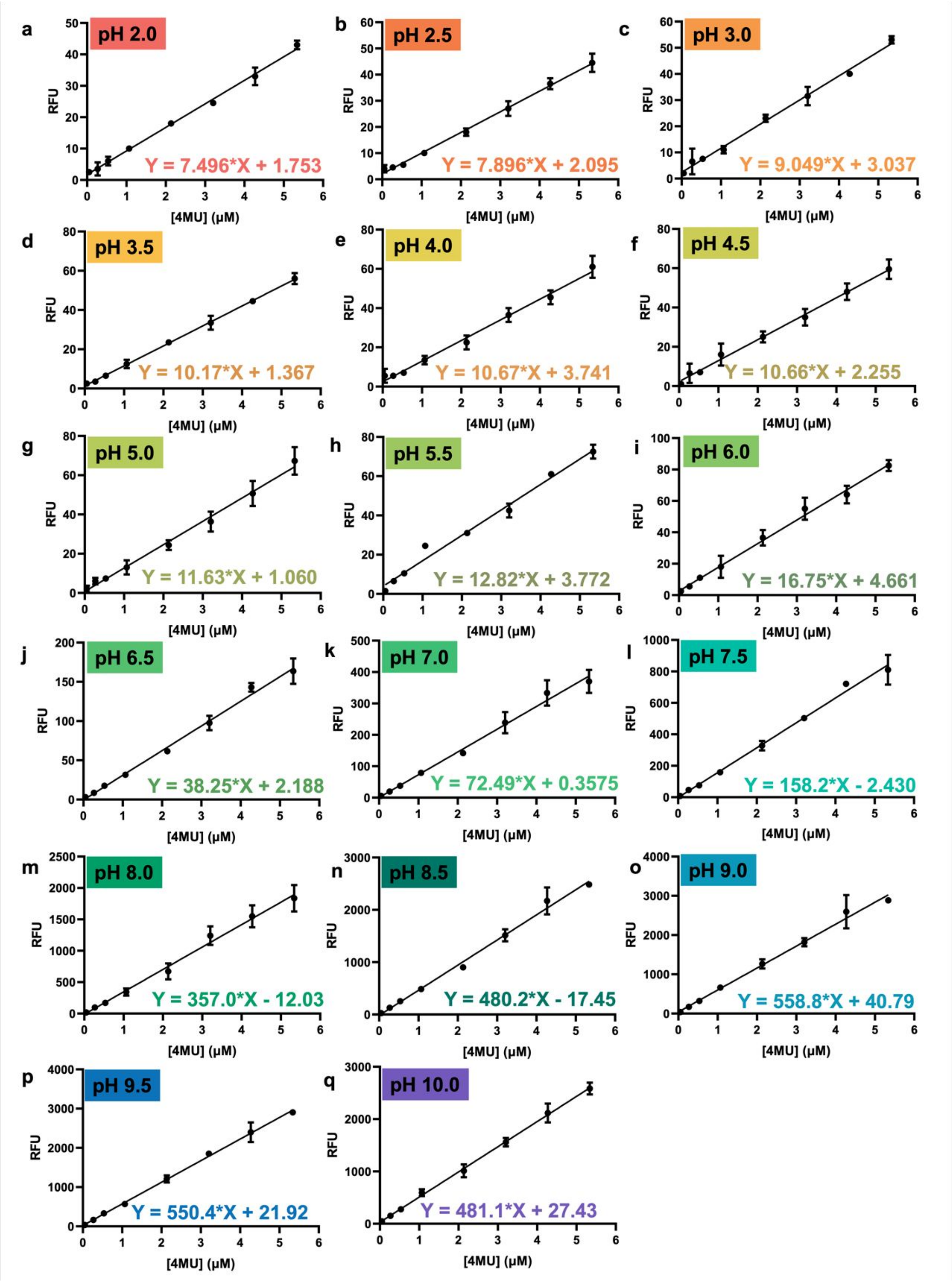

Figure S1. The standard curves of 4MU in the universal buffer at various pHs. Curves **a** to **q** fitted by linear regression.

All the standard curves were plotted by monitoring the fluorescence from compound 4MU at λ_{ex} = 360 nm and λ_{em} = 450 nm in a BMG Fluostar fluorescence microplate reader with gain 1200, in the buffer of 25 mM Bis-tris propane, 25 mM citrate, and 300 mM NaCl, pH 2.0 - 10.0, titrated by HCl. Standard curve equations for 4MU fluorescence at various pHs are listed in **Supplementary Table 2**.

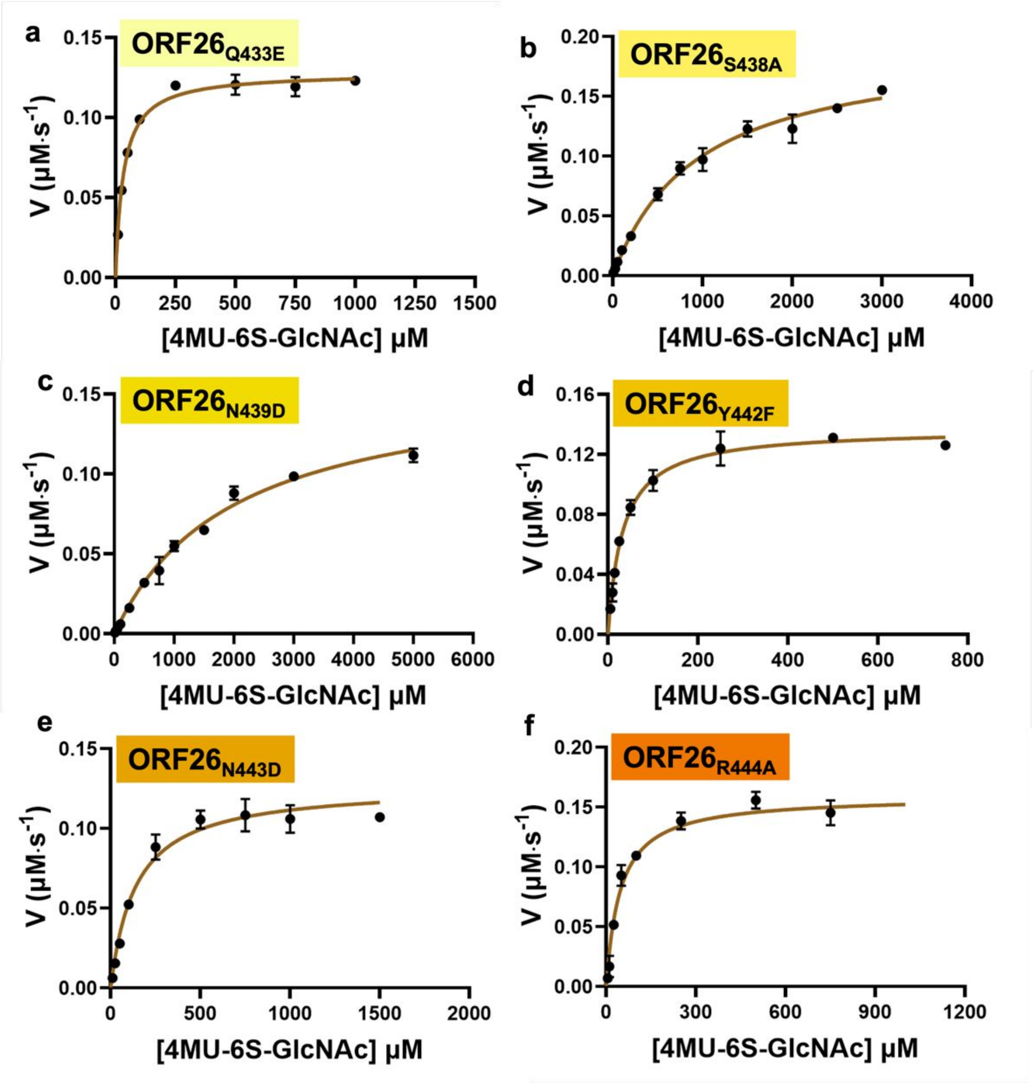

Figure S2. Michaelis-Menten plots for hydrolysis of 4MU-6S-GlcNAc by F3-ORF26 variants at pH 6.0.

(**a**) 2 nM ORF26$_{Q433E}$ hydrolyzes 10 μM to 1000 μM substrate. (**b**) 2 nM ORF26$_{S438A}$ hydrolyzes 5 μM to 3000 μM substrate. (**c**) 4 nM ORF26$_{N439D}$ hydrolyzes 10 μM to 5000 μM substrate. (**d**) 2 nM ORF26$_{Y442F}$hydrolyzes 10 μM to 1000 μM substrate. (**e**) 2 nM ORF26$_{N443D}$ hydrolyzes 10 μM to 1500 μM substrate. (**f**) 2 nM ORF26$_{R444A}$ hydrolyzes 5 μM to 750 μM substrate.

a F3-ORF26 (query with GH20 domain), AST178 (± 50% identity), No fragment

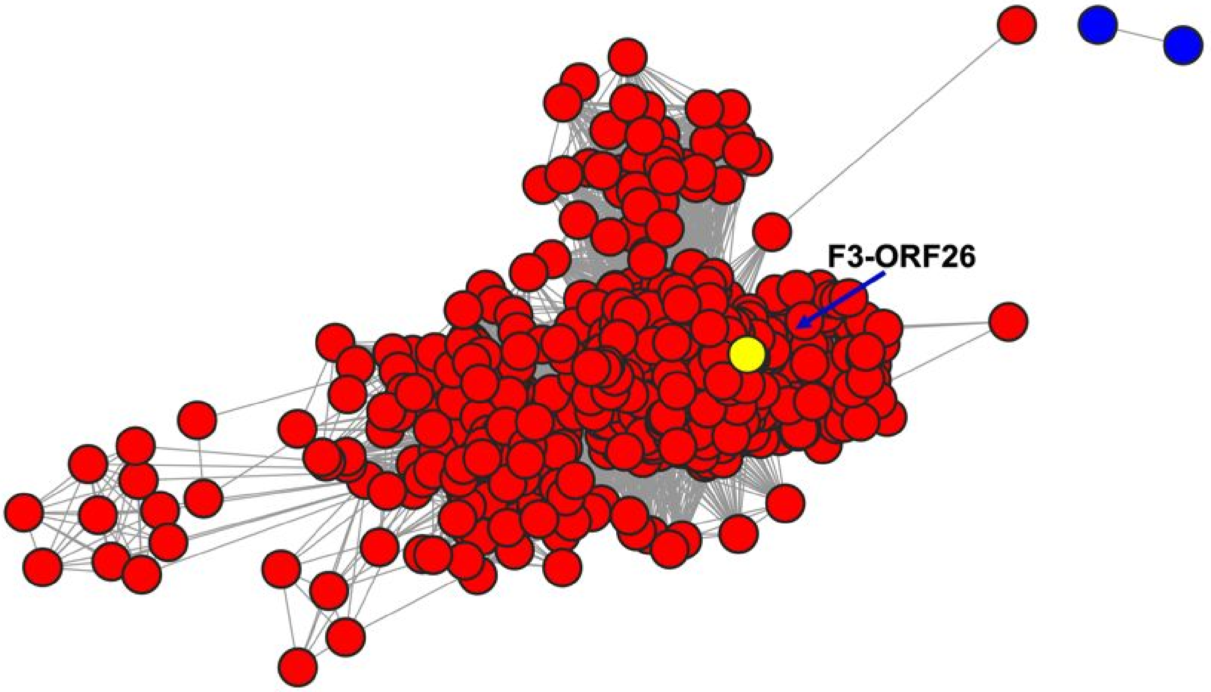

b F3-ORF26 (query with GH20 domain), AST202 (± 53% identity), No fragment

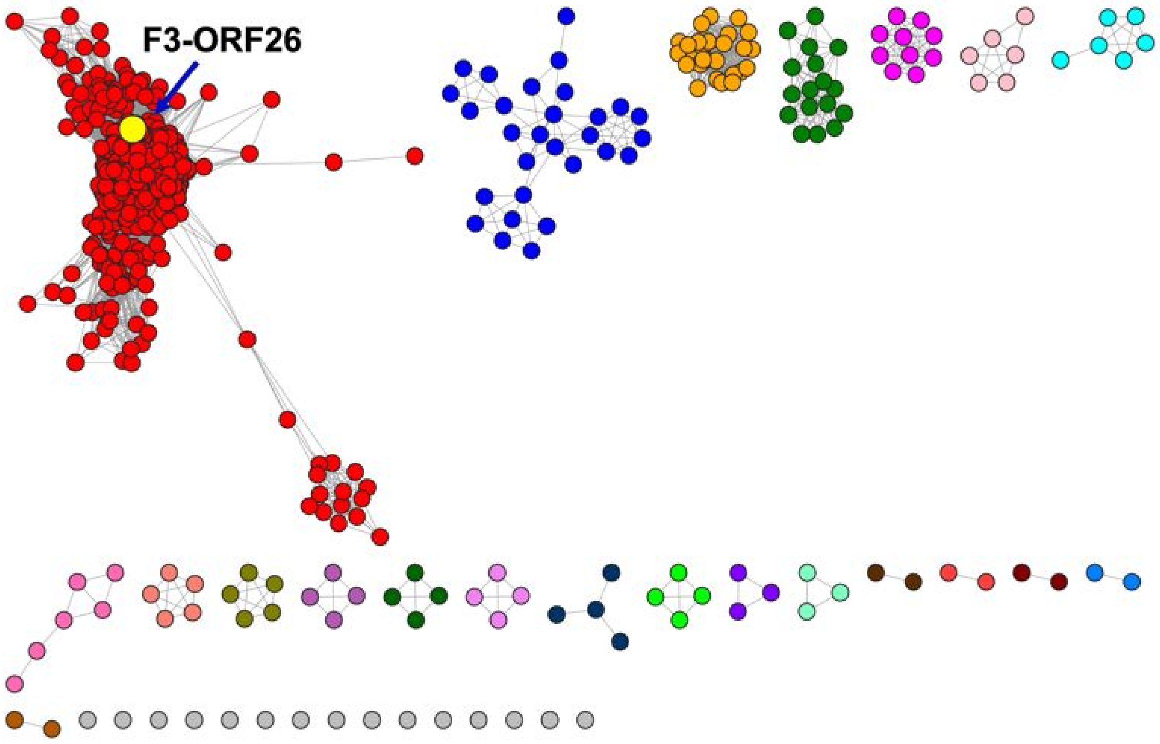

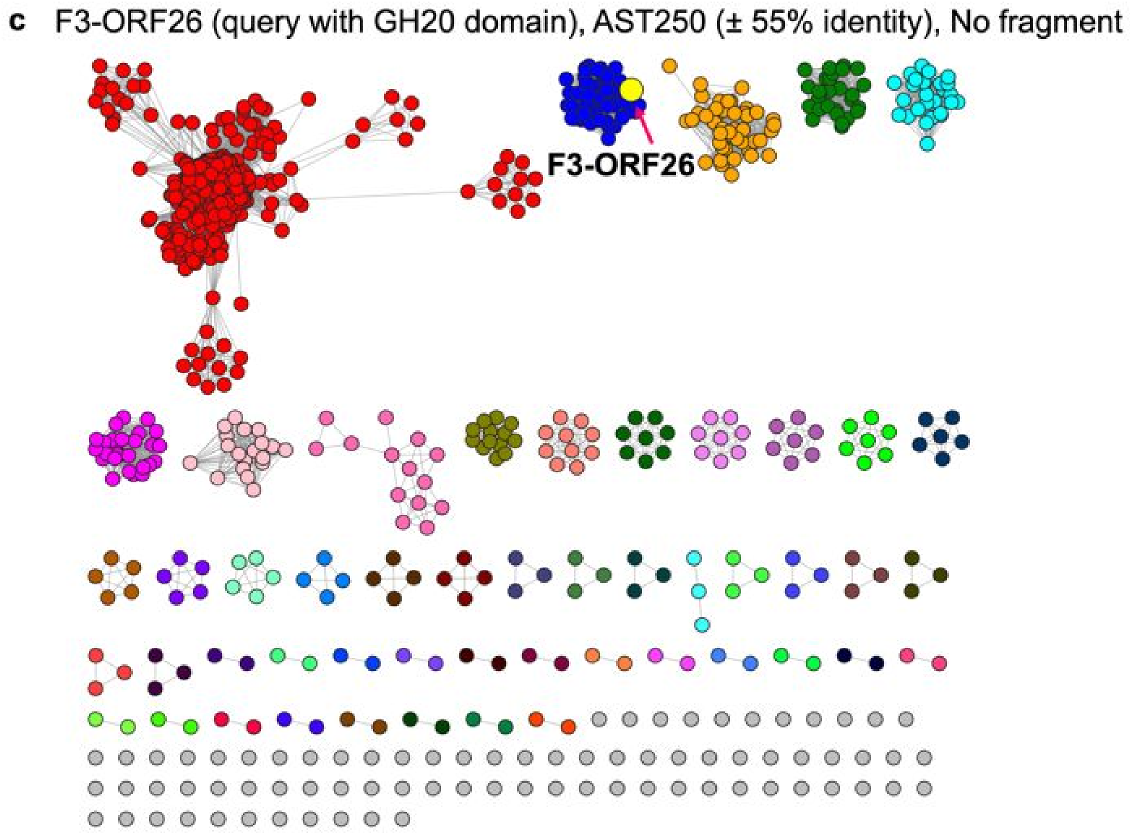

Figure S3. F3-ORF26 6-sulfo-β-GlcNAcases (highlighted as a yellow dot) mapped on the focused Uniport SSN created for the GH20 domain.

The starting setting AST178 is not high enough to separate the subgroups. Increasing the AST thresholds from (**a**) 178, (**b**) 202 to (**c**) 250 leads to the separation of subclusters that separate F3-ORF26 type sequences from others.

a

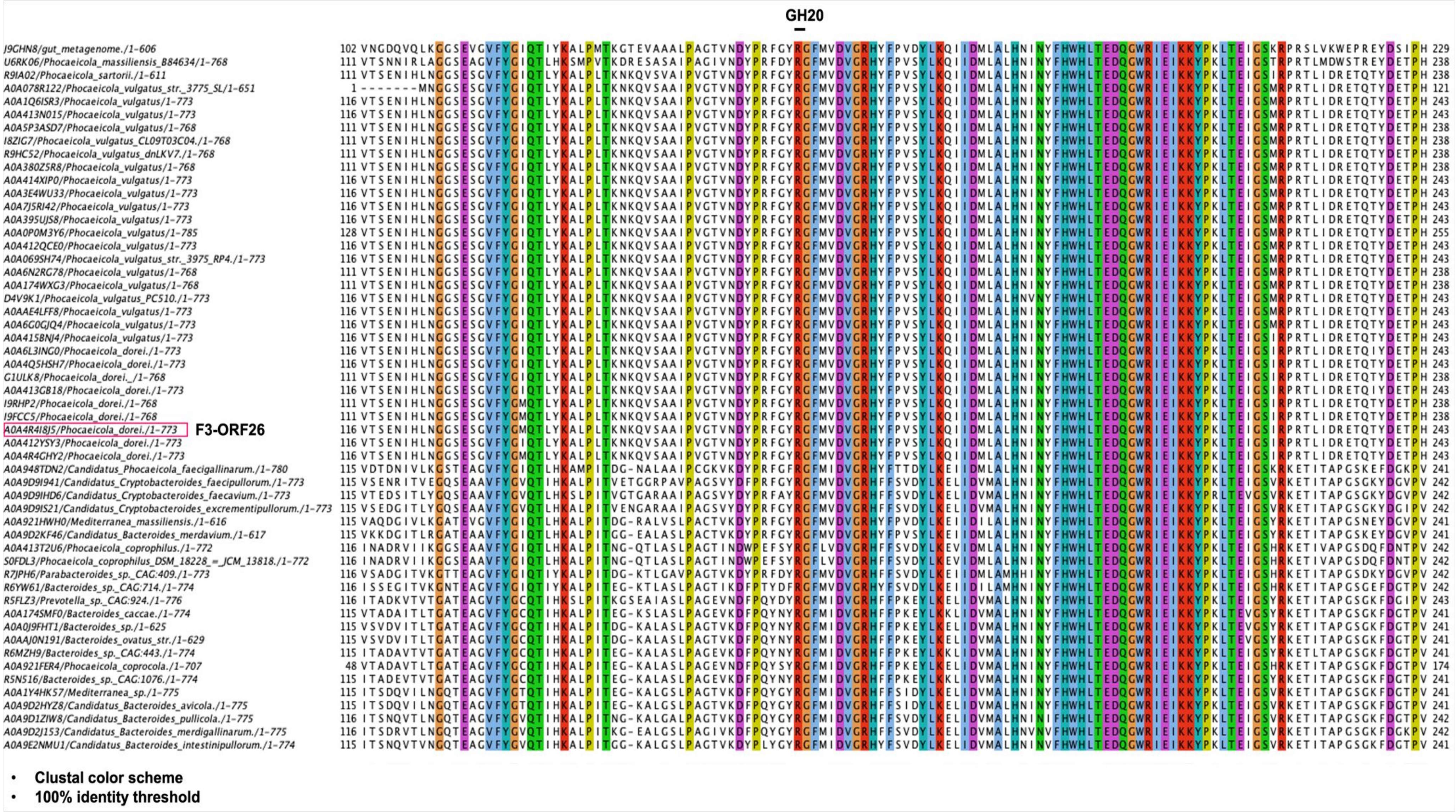
GH20
F3-ORF26
A0A4R4I8J5/Phocaeicola_dorei./1-773
• Clustal color scheme
• 100% identity threshold

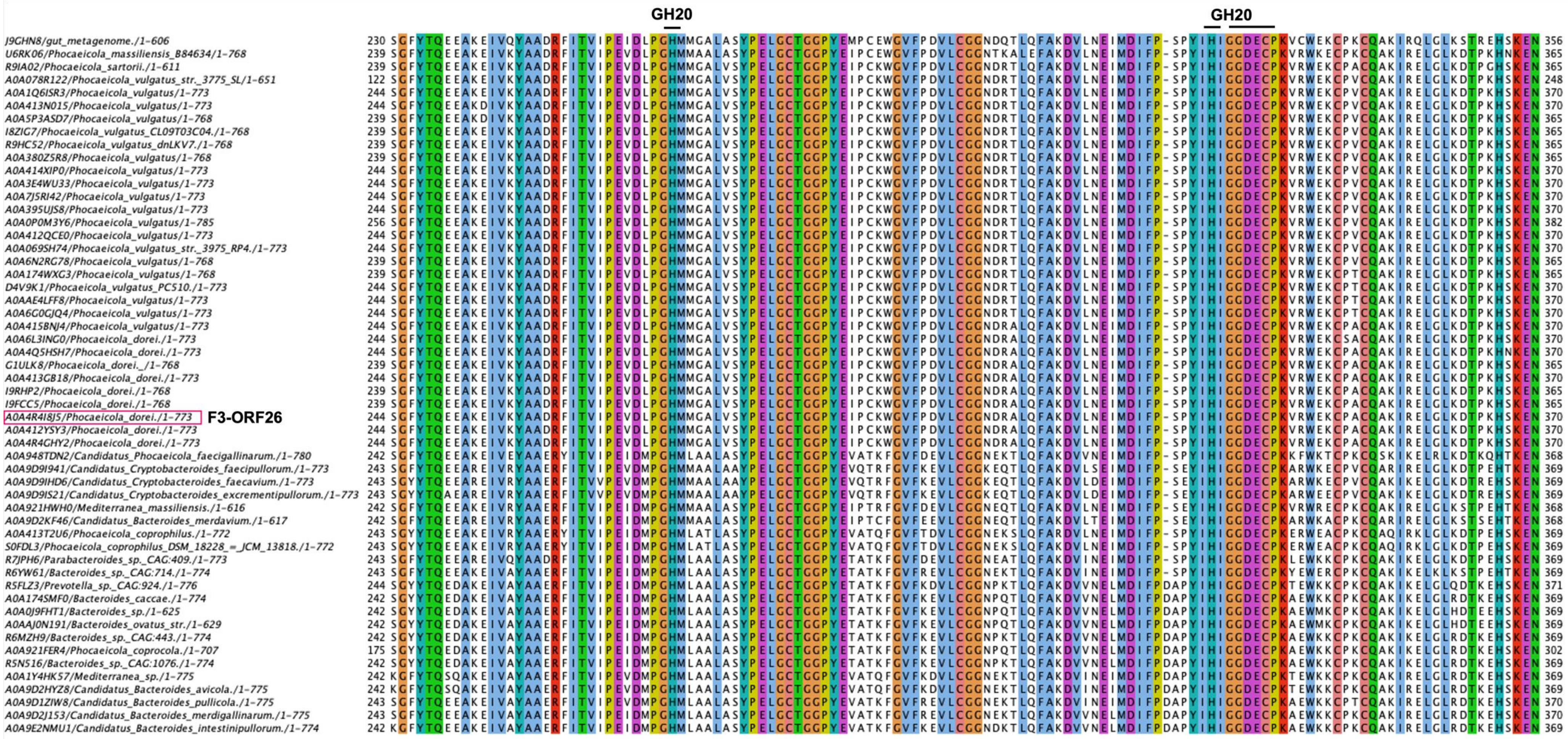

- **Clustal color scheme**
- **100% identity threshold**

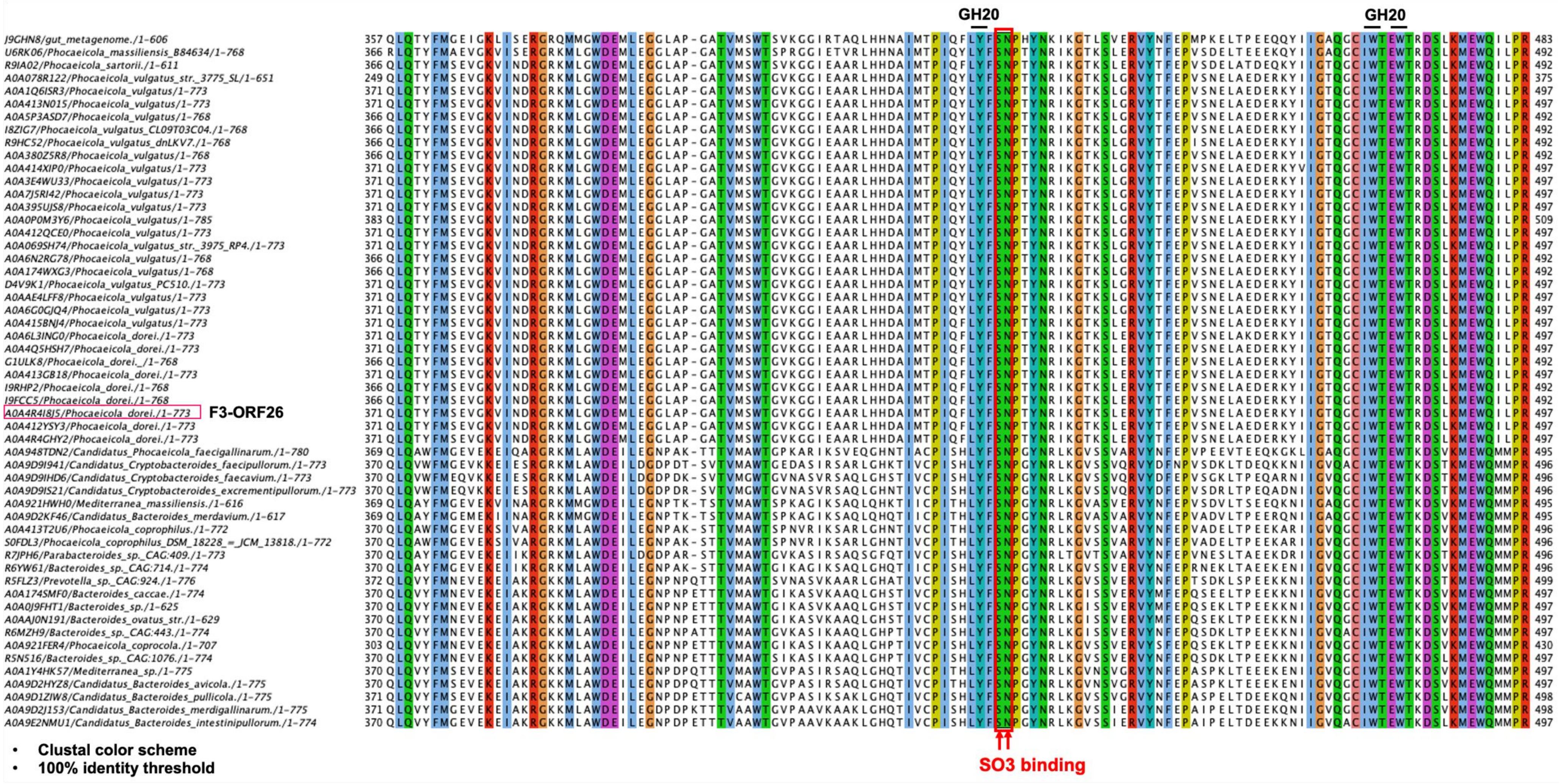
GH20
GH20
F3-ORF26
SO3 binding
• Clustal color scheme
• 100% identity threshold

b

Sequence Cluster 2 / Node Cluster 2

Full Sequences

Number of IDs: UniProt: 59

MSA **PNG**

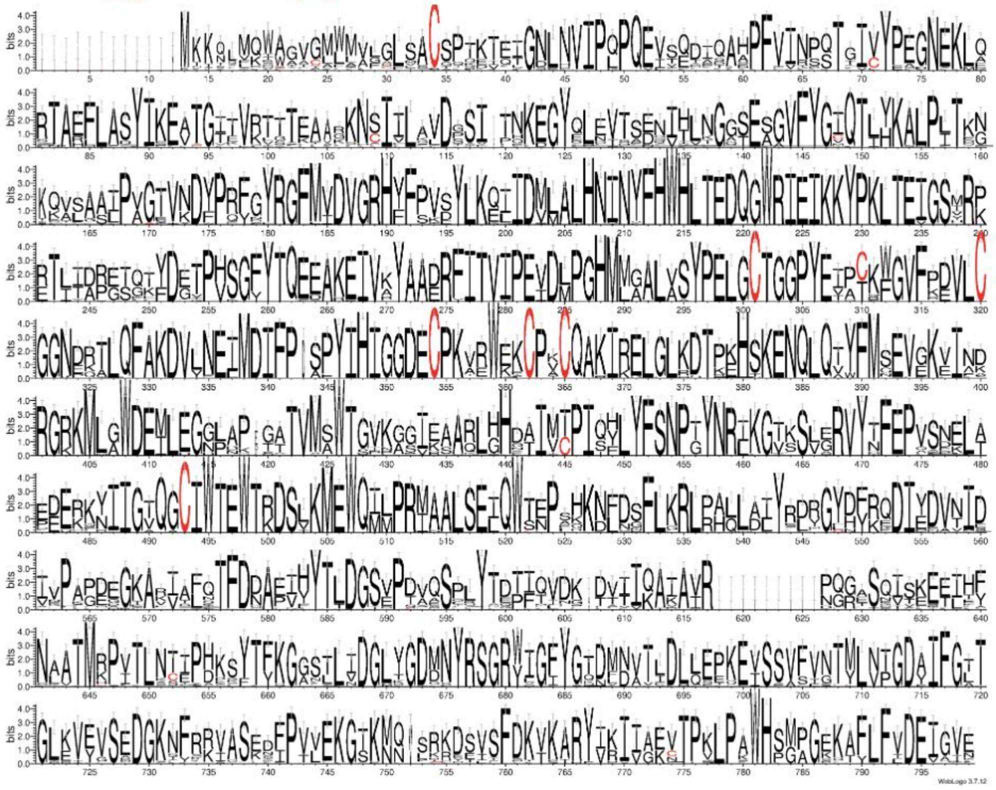

Figure S4 (**a**) Multiple sequence alignment (MSA) and (**b**) sequence logo highlighting the conserved sulfate-binding feature in the F3-ORF26 protein cluster (cluster 2 in blue in Supplementary Figure 8c).

The key conserved residues known to be involved in classical GH20 activity, including the catalytic D-E amino acid pair, and the sulfate-recognizing residues are highlighted.

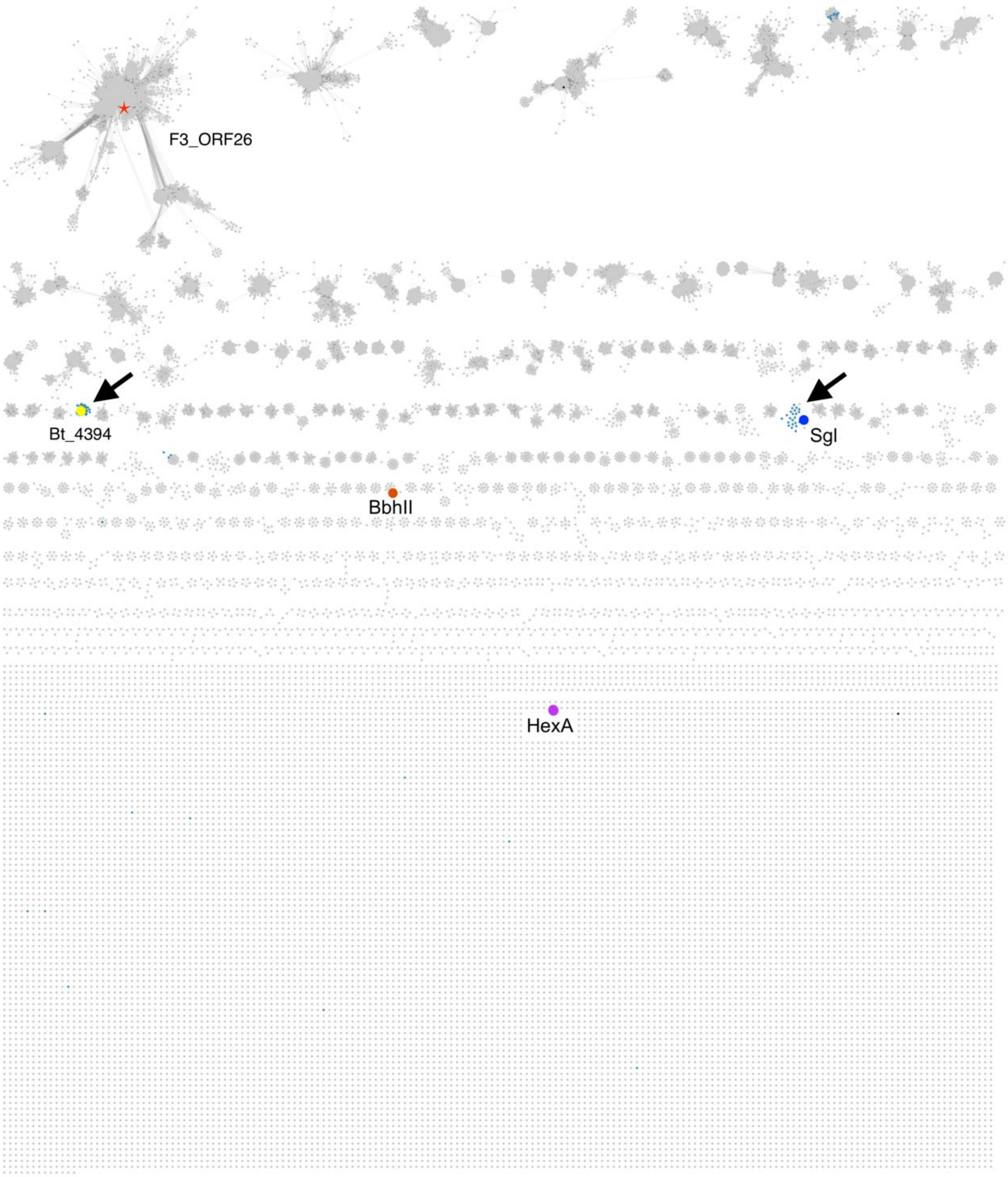

Figure S5. Full-resolution Sequence Similarity Network (SSN) for the GH20 Domain with an Alignment Score Threshold (AST) of 130 (PF00728, Domain, no fragments).

Previously characterised sequences are identified with colored dots: Q5MAH5 (sgl from *Prevotella* sp. RS2), D4QAP5 (BbhII from *Bifidobacterium bifidum*), Q89ZI3 (BT_4394 from *Bacteroides thetaiotaomicron*, P06865 (HexA from *Homo sapiens*) and A0A4R4I8J5 (as red star, ORF26 from *Phocaeicola dorei*). Sequences identified in blue contain the key motif YYI**NR**. Sequences in clusters identified with arrows (clusters containing Bt_4394 and Sgl) have key motif YYINR in the sequences at locations suitable for substrate binding through MSA.

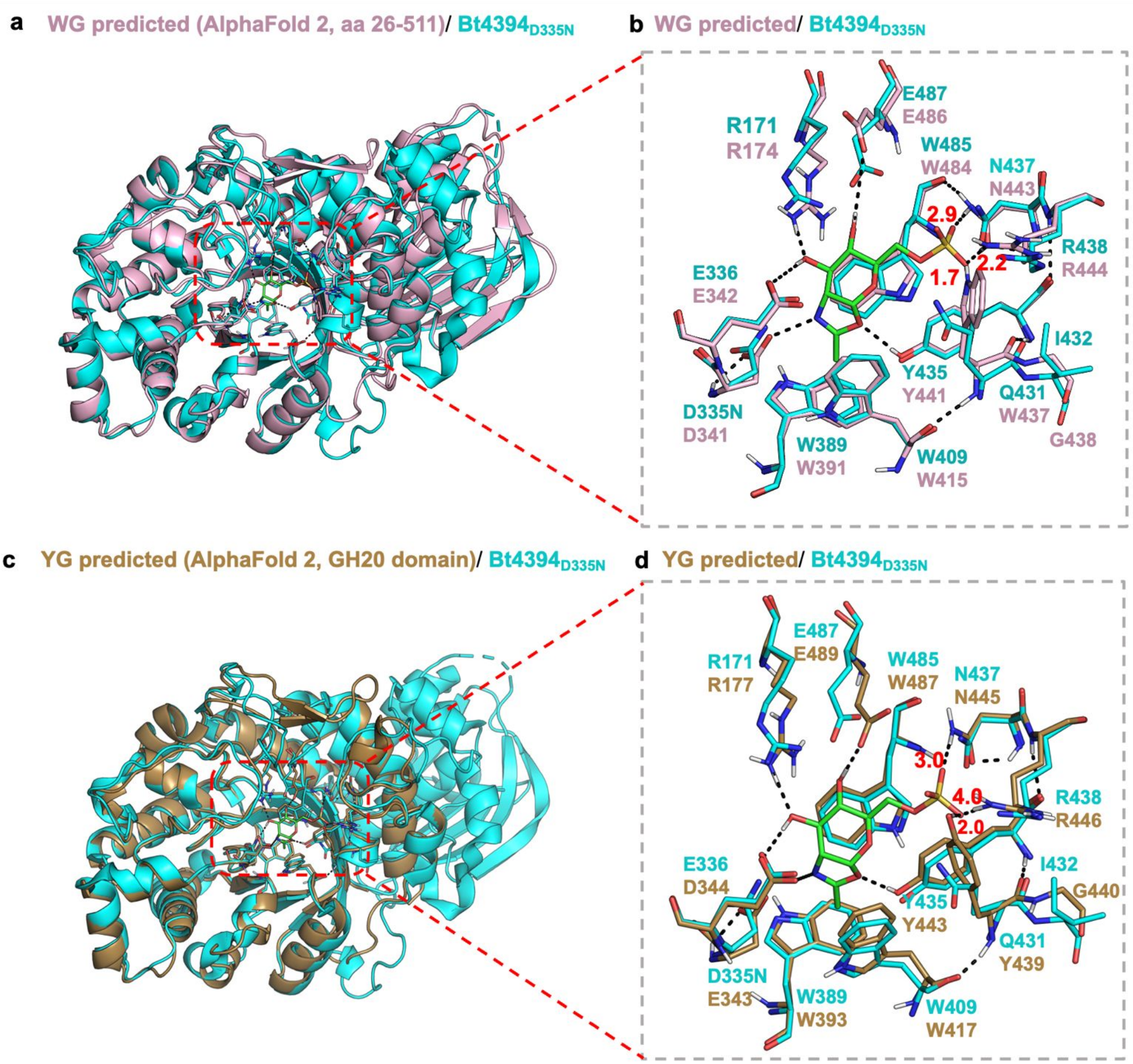

Figure S6. The WG_{WT} and YG_{WT} structure predicted by AlphaFold 2, and its GH20 catalytic domain aligned with the $Bt4394_{D335N}$-6S-NAG-oxazoline intermediate (PDB: 7DVB). The 6S-NAG-oxazoline ligand from PDB:7DVB is in green. H-bond distances are for donor-to-acceptor in Å.

(**a**) Overlay of the structures of $Bt4394_{D335N}$-6S-NAG-oxazoline intermediate complex (cyan) and the predicted unrelaxed apo WG_{WT} (dark pink) predicted by AlphaFold 2. (**b**) Zoom-in view of the aligned active site, showing polarizing residue D341 and the general acid/base residue E342 in WG, aligned well with D335N and E336 in Bt4394. Additionally, R174 and E486 in WG, which coordinate the 3', 4'-OH groups, overlaid well with R171 and E487 in Bt4394. At the sulfate binding site, N443 and R444 in WG, aligned well with N437 and R438 in Bt4394.

(**c**) Overlay of the structures of $Bt4394_{D335N}$-6S-NAG-oxazoline intermediate complex (cyan) and the predicted unrelaxed apo YG_{WT} (khaki) predicted by AlphaFold 2. (**d**) Zoom-in view of the aligned active site, showing polarizing residue D343 and the general acid/base residue E344 in YG aligned well with D335N and E336 in Bt4394. Around the 3', 4'-OH binding site, R177 and E489 in YG, overlaid well with R171 and E487 in Bt4394. Meanwhile, N443 and R444 at sulfate binding site in YG_{WT}, aligned well with N437 and R438 in Bt4394.

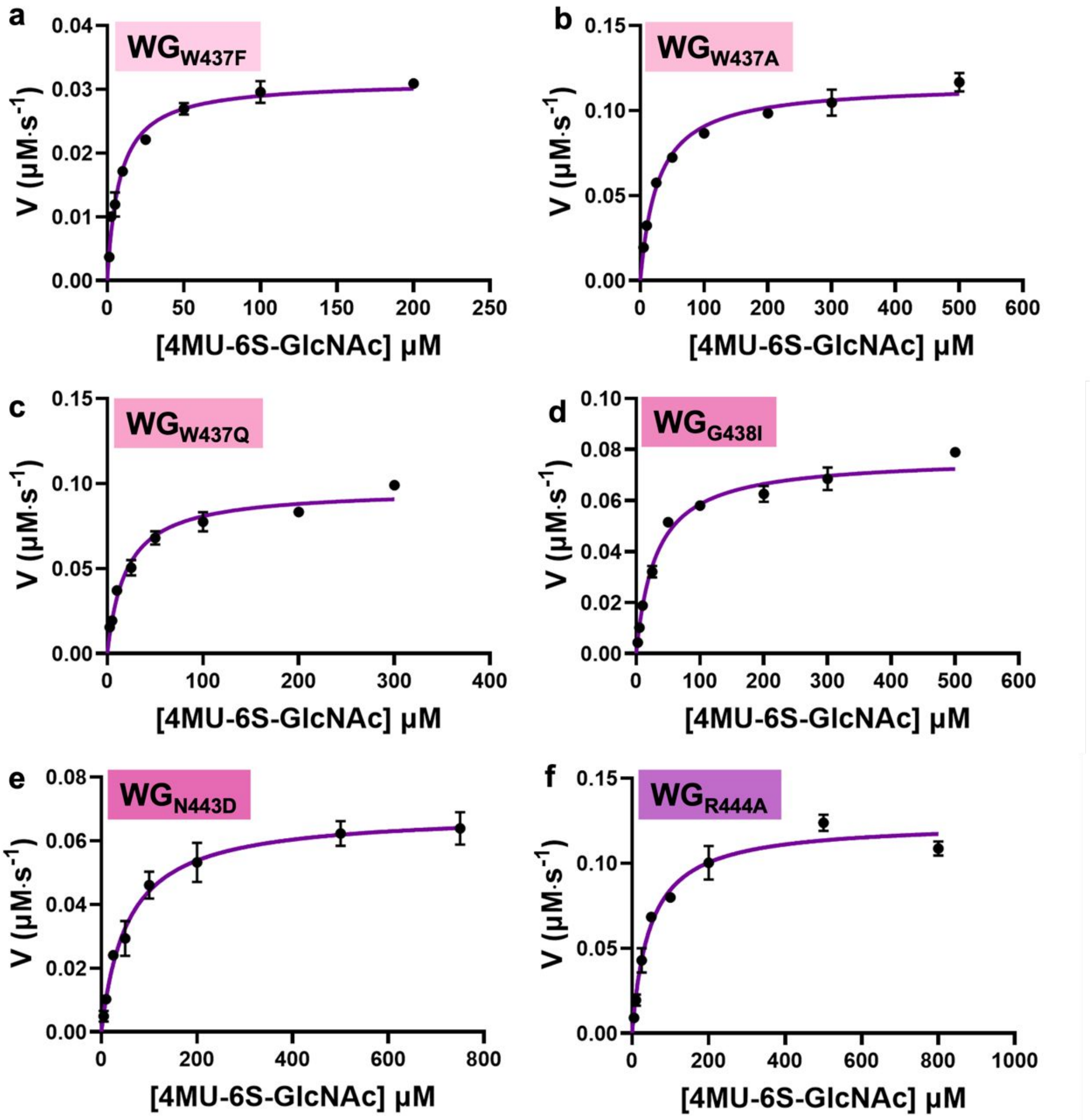

Figure S7. Michaelis-Menten plots for hydrolysis of 4MU-6S-GlcNAc by WG variants at pH 6.0.

(**a**) 0.5 nM WG_{W437F} variant hydrolyzes 1.25 μM to 200 μM substrates. (**b**) 2 nM WG_{W437A} variant hydrolyzes 2.5 μM to 300 μM substrates. (**c**) 2 nM WG_{W437Q} variant hydrolyzes 2.5 μM to 300 μM substrates. (**d**) 2 nM WG_{G438I} variant hydrolyzes 2.5 μM to 500 μM substrates. (**e**) 1 nM WG_{N443D} variant hydrolyzes 5 μM to 750 μM substrates. (**f**) 2 nM WG_{R444A} variant hydrolyzes 5 μM to 800 μM substrates.

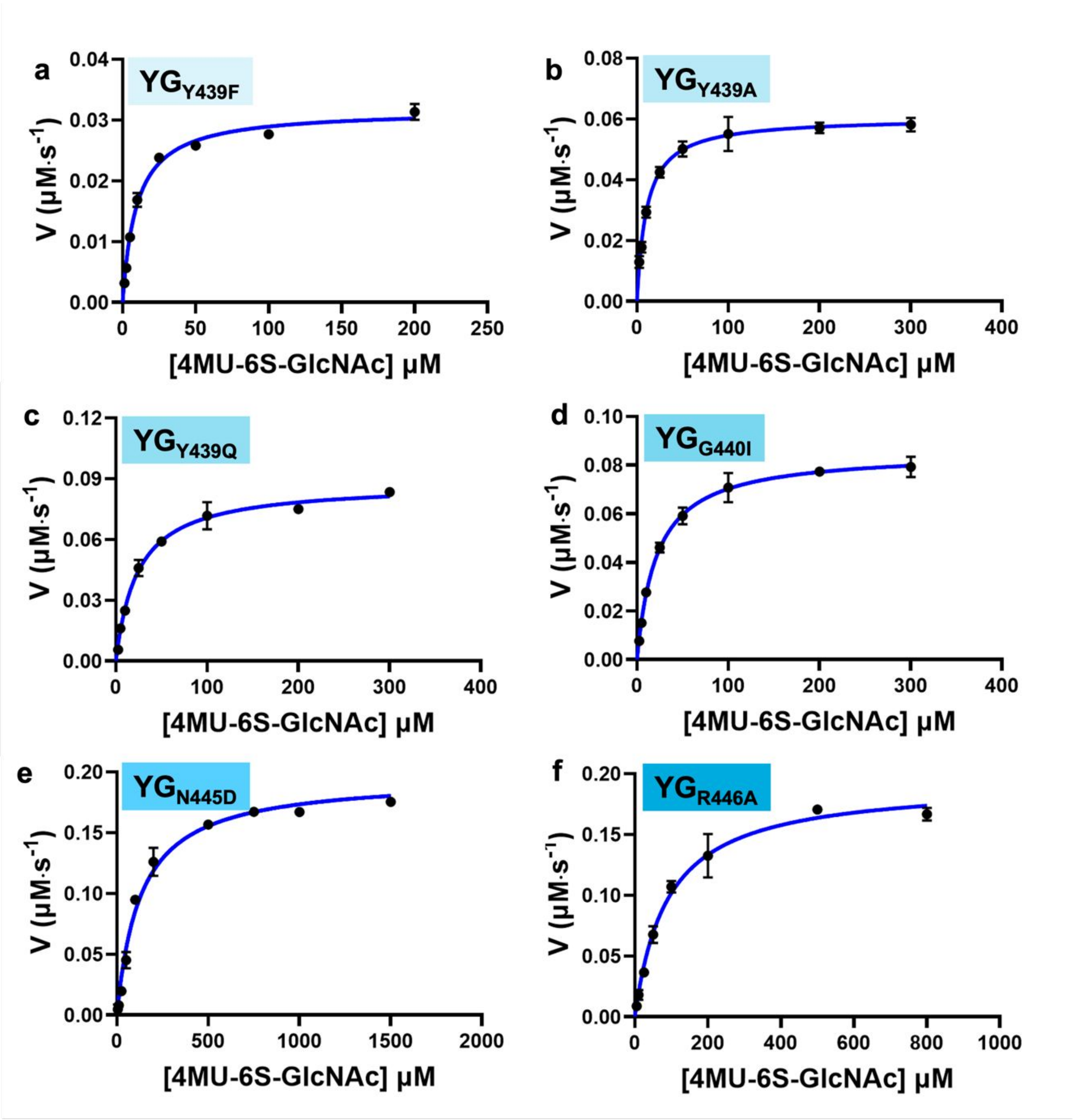

Figure S8. Michaelis-Menten plots for hydrolysis of 4MU-6S-GlcNAc by YG variants at pH 6.0.

(**a**) 0.46 nM YG_{Y439F} mutant hydrolyzes 1.25 µM to 200 µM substrates. (**b**) 2 nM YG_{Y439A} variant hydrolyzes 2.5 µM to 300 µM substrates. (**c**) 2 nM YG_{Y439Q} variant hydrolyzes 2.5 µM to 300 µM substrates. (**d**) 2 nM YG_{G440I} variant hydrolyzes 2.5 µM to 300 µM substrates. (**e**) 2 nM YG_{N445D} variant hydrolyzes 2.5 µM to 300 µM substrates. (**f**) 2 nM YG_{R446A} variant hydrolyzes 5 µM to 800 µM substrates.

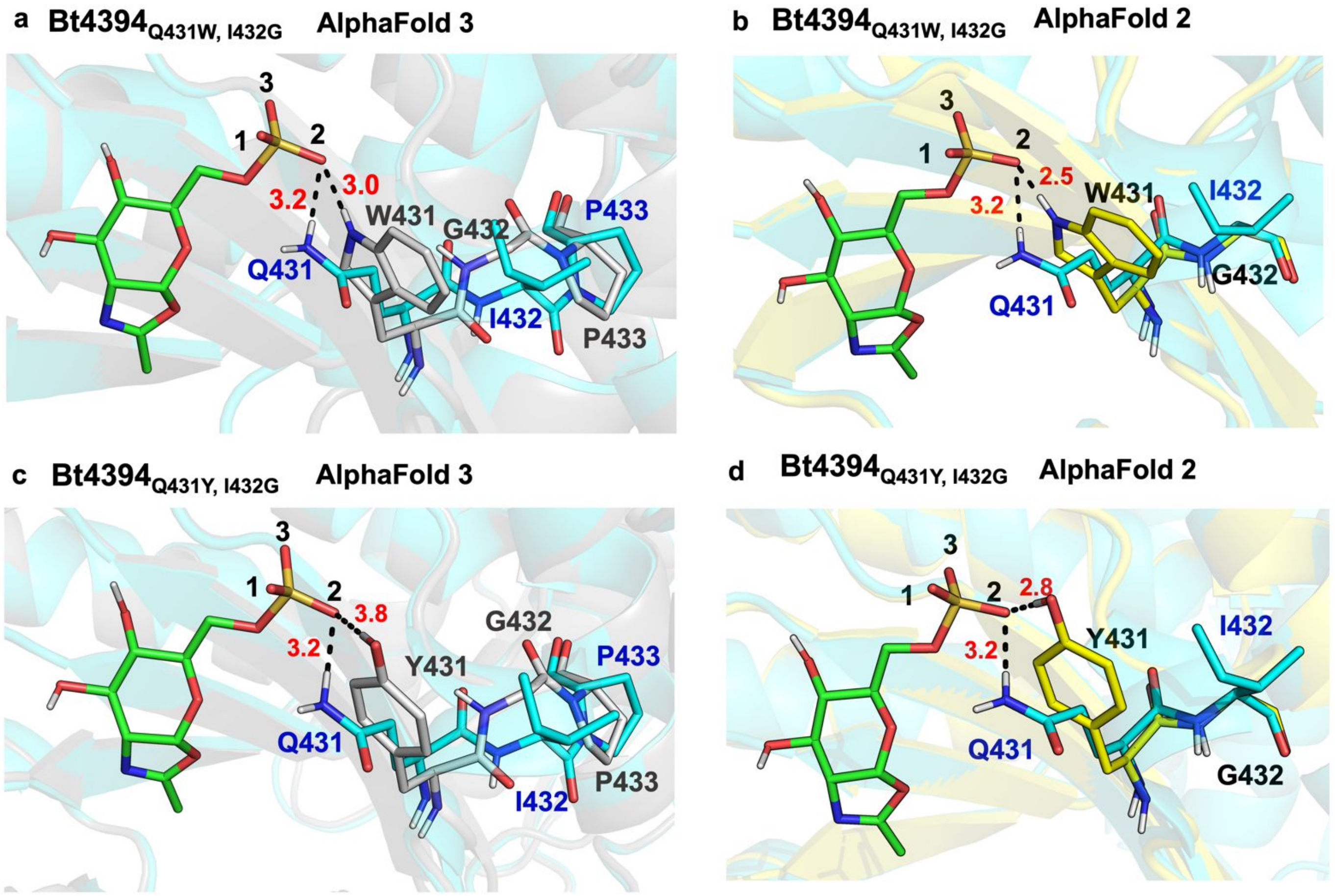

Figure S9. Overlay the structures of the 6S-NAG-oxazoline intermediate complexes of Bt4394$_{D335N}$ (PDB: 7DVB, in cyan) in comparison with the Bt4394$_{Q431W,\ I432G}$ generated by (**a**) AlphaFold 3 and (**b**) AlphaFold 2. Bt4394$_{D335N}$ (PDB: 7DVB, in cyan) in comparison with the Bt4394$_{Q431Y,\ I432G}$ structures generated by (**c**) AlphaFold 3 and (**d**) AlphaFold 2 with the default settings. The 6S-NAG-oxazoline ligand from PDB:7DVB is in green. H-bond distances are for donor-to-acceptor in Å.

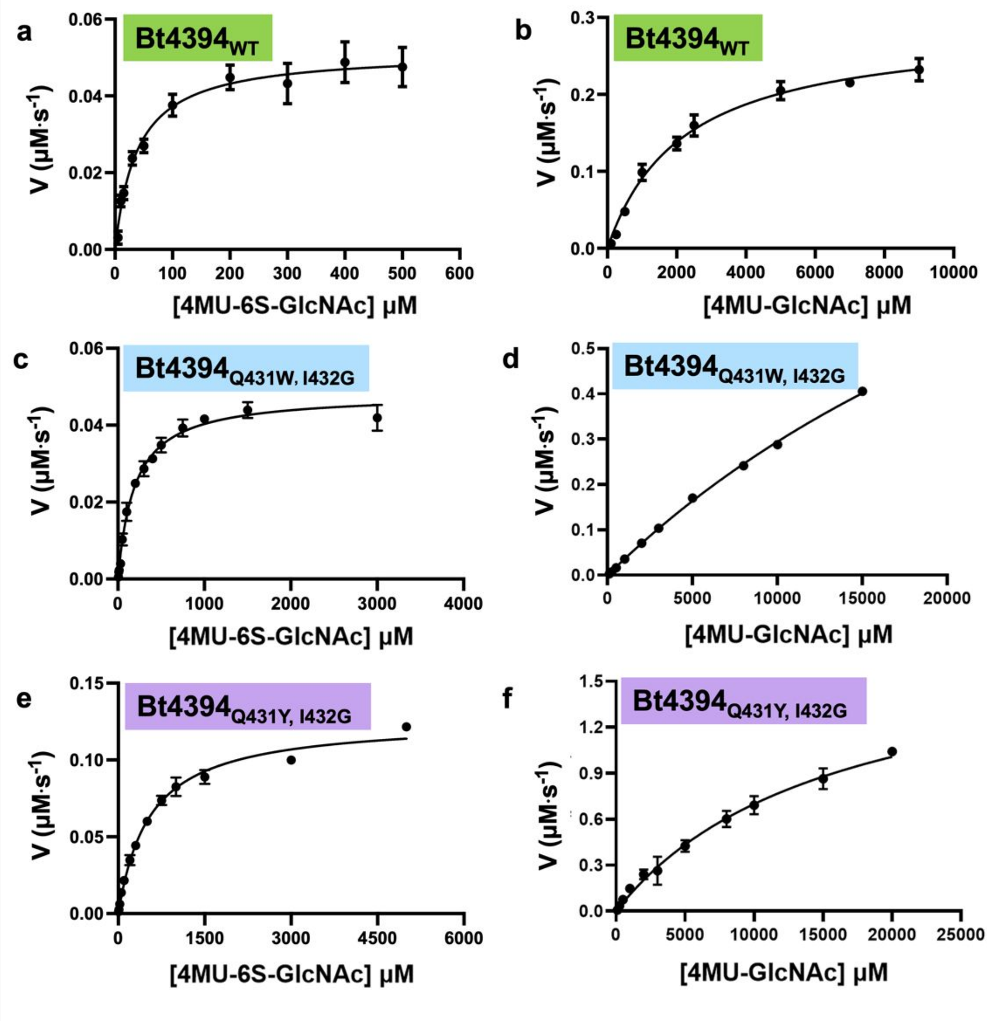

Figure S10. Michaelis-Menten plots for illustrating substrate specificity of sulfated substrate 4MU-6S-GlcNAc and non-sulfated 4MU-GlcNAc with $Bt4394_{WT}$ and its variants $Bt4394_{Q431W,I432G}$ and $Bt4394_{Q431Y,\ I432G}$ at pH 5.5.

(**a**) 2 nM $Bt4394_{WT}$ hydrolyzes 5 µM to 500 µM 4MU-6S-GlcNAc substrate. (**b**) 100 nM $Bt4394_{WT}$ hydrolyzes 100 µM to 9000 µM 4MU-GlcNAc substrate. (**c**) 4 nM $Bt4394_{Q431W,I432G}$ hydrolyzes 5 µM to 3000 µM 4MU-6S-GlcNAc substrate. (**d**) 0.48 µM $Bt4394_{Q431W,I432G}$ hydrolyzes 100 µM to 15000 µM 4MU-GlcNAc substrate. (**e**) 4 nM $Bt4394_{Q431Y,I432G}$ hydrolyzes 5 µM to 5000 µM 4MU-6S-GlcNAc substrate. (**f**) 0.48 µM $Bt4394_{Q431Y,I432G}$ hydrolyzes 100 µM to 20000 µM 4MU-GlcNAc substrate.

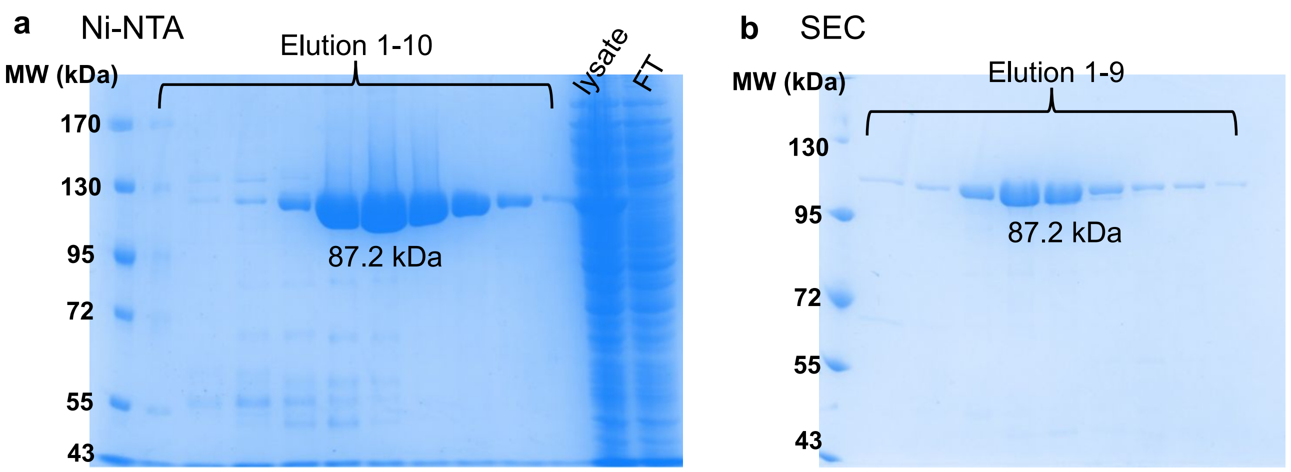

Figure S11. SDS-PAGE gels for F3-ORF26 protein purification.

(**a**) Ni-NTA affinity column purification. (**b**) Size exclusion column purification.

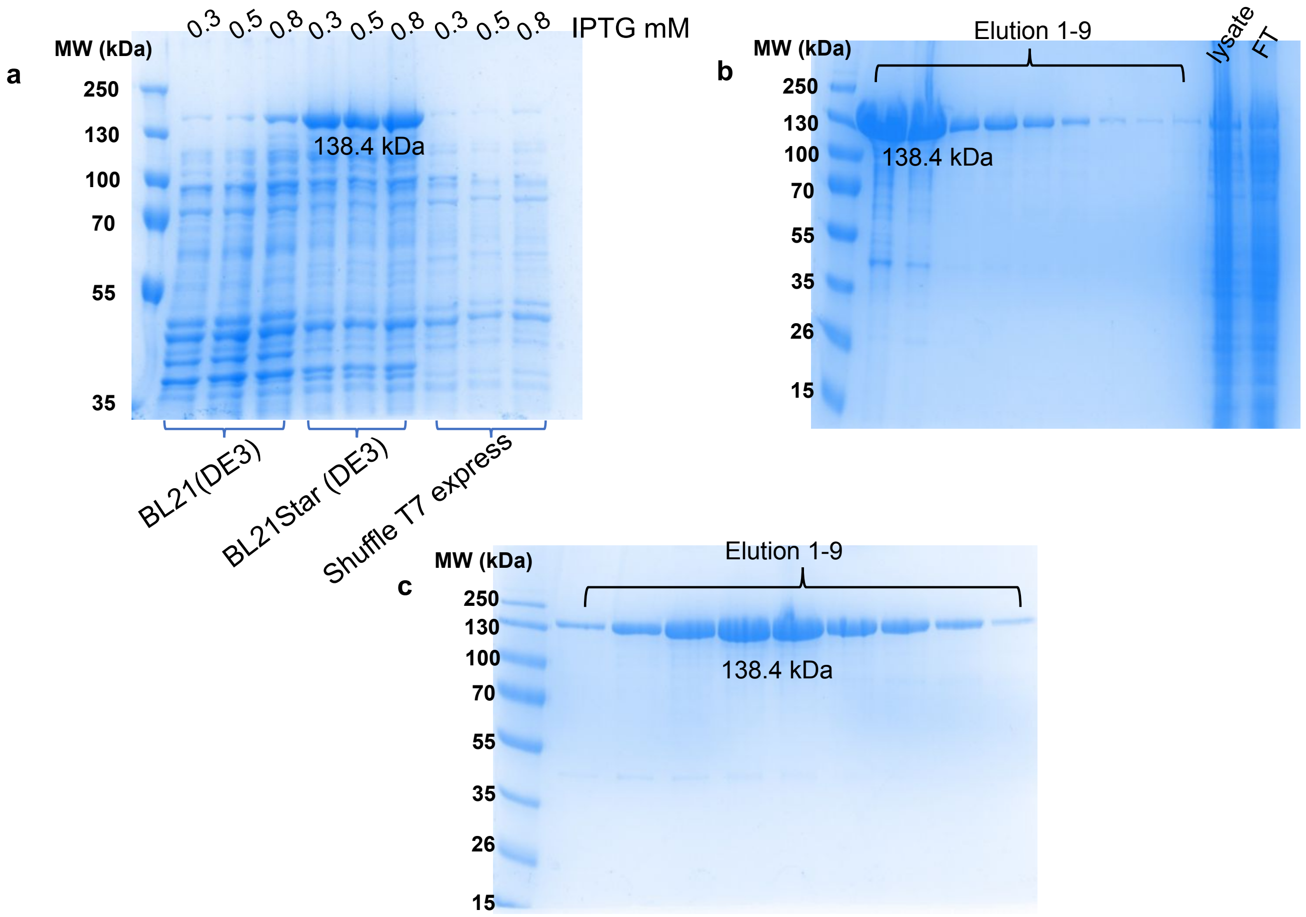

Figure S12. SDS-PAGE gels for expression and purification of WG protein.

(**a**) Expression test of WG protein. (**b**) Ni-NTA affinity column purification. (**c**) Size exclusion column purification.

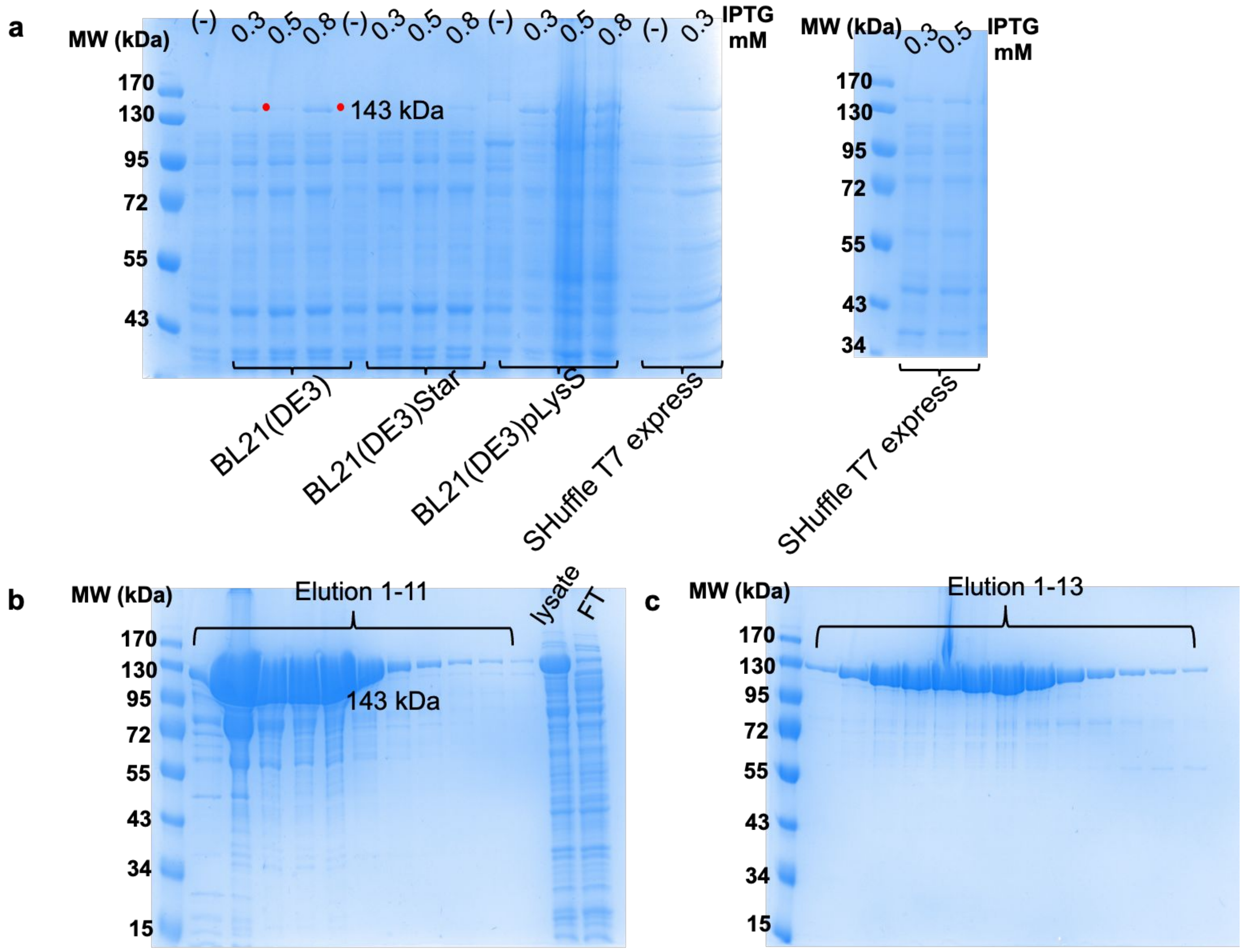

Figure S13. SDS-PAGE gels for expression and purification of YG protein.

(**a**) Expression test of YG protein. (**b**) Ni-NTA affinity column purification. (**c**) Size exclusion column purification.

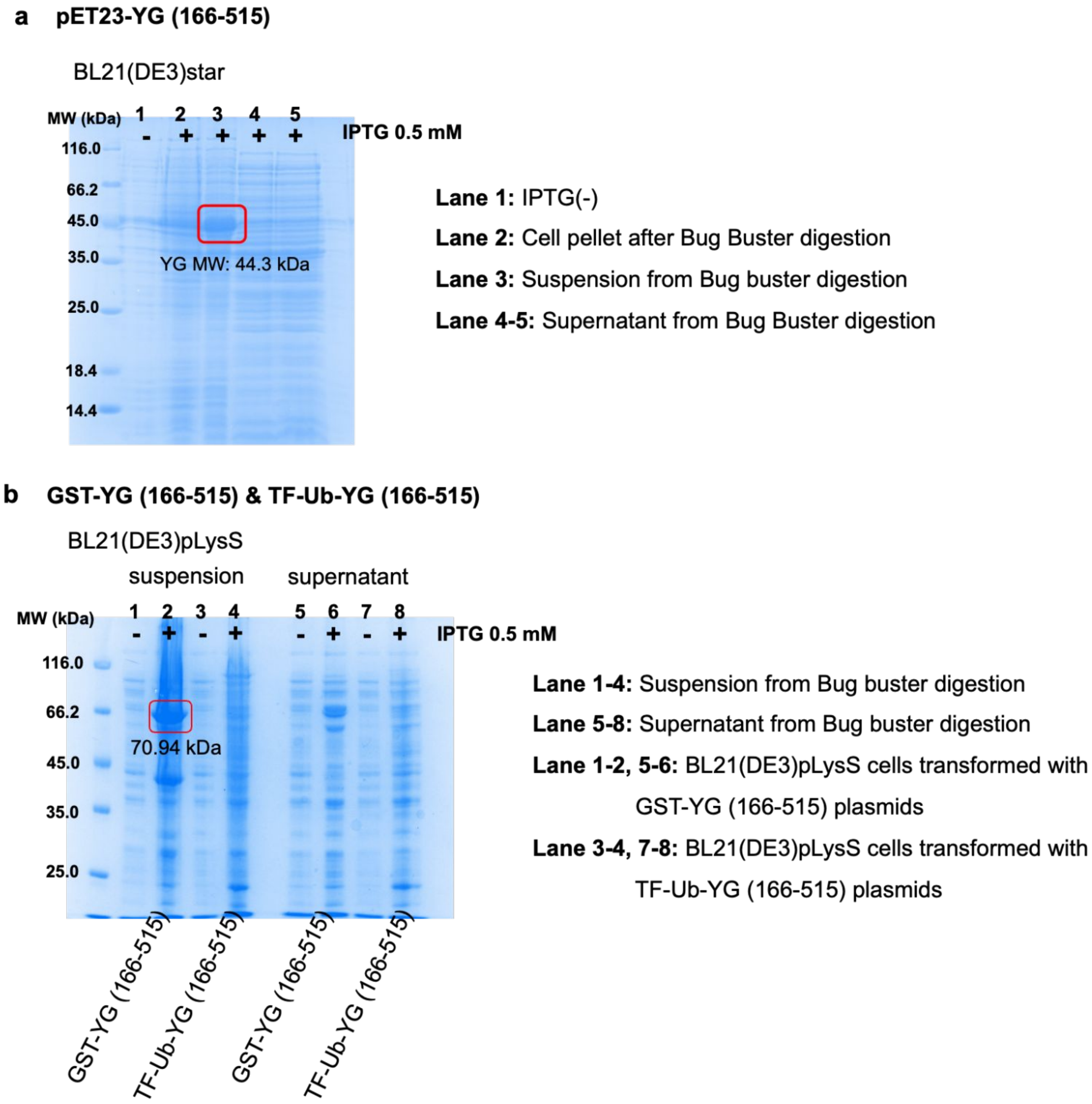

Figure S14. SDS-PAGE gels for expression test of truncated YG protein.

(**a**) Expression test of truncated YG protein plasmids pET23-YG(166-515)-6His expressed in the BL21(DE3)Star cells. The truncated YG protein is insoluble and forms the inclusion body in the cell pellet. (**b**) Expression test of GST-tagged and TF-Ub-tagged truncated YG protein plasmids pGEX2T-YG(166-515)-6His and pET28a-TF-YG(166-515)-6His expressed in the BL21(DE3)pLysS cell. Similarly, The GST-tagged and TF-Ub-tagged truncated YG proteins are insoluble and form the inclusion body, the supernatant fraction (Lane 6) was then purified by Ni-NTA spin column. Unfortunately, no soluble protein was eluted.
